# Biologically Based Methods for Control of Fumonisin-Producing *Fusarium* Species and Reduction of the Fumonisins

**DOI:** 10.3389/fmicb.2016.00548

**Published:** 2016-04-26

**Authors:** Johanna F. Alberts, Willem H. van Zyl, Wentzel C. A. Gelderblom

**Affiliations:** ^1^Mycotoxicology and Chemoprevention Research Group, Institute of Biomedical and Microbial Biotechnology, Cape Peninsula University of TechnologyBellville, South Africa; ^2^Microbiology Department, Stellenbosch UniversityStellenbosch, South Africa

**Keywords:** *Fusarium*, fumonisins, prevention, biological control, reduction, sub-Saharan countries

## Abstract

Infection by the fumonisin-producing *Fusarium* spp. and subsequent fumonisin contamination of maize adversely affect international trade and economy with deleterious effects on human and animal health. In developed countries high standards of the major food suppliers and retailers are upheld and regulatory controls deter the importation and local marketing of fumonisin-contaminated food products. In developing countries regulatory measures are either lacking or poorly enforced, due to food insecurity, resulting in an increased mycotoxin exposure. The lack and poor accessibility of effective and environmentally safe control methods have led to an increased interest in practical and biological alternatives to reduce fumonisin intake. These include the application of natural resources, including plants, microbial cultures, genetic material thereof, or clay minerals pre- and post-harvest. Pre-harvest approaches include breeding for resistant maize cultivars, introduction of biocontrol microorganisms, application of phenolic plant extracts, and expression of antifungal proteins and fumonisin degrading enzymes in transgenic maize cultivars. Post-harvest approaches include the removal of fumonisins by natural clay adsorbents and enzymatic degradation of fumonisins through decarboxylation and deamination by recombinant carboxylesterase and aminotransferase enzymes. Although, the knowledge base on biological control methods has expanded, only a limited number of authorized decontamination products and methods are commercially available. As many studies detailed the use of natural compounds *in vitro*, concepts in reducing fumonisin contamination should be developed further for application *in planta* and in the field pre-harvest, post-harvest, and during storage and food-processing. In developed countries an integrated approach, involving good agricultural management practices, hazard analysis and critical control point (HACCP) production, and storage management, together with selected biologically based treatments, mild chemical and physical treatments could reduce fumonisin contamination effectively. In rural subsistence farming communities, simple, practical, and culturally acceptable hand-sorting, maize kernel washing, and dehulling intervention methods proved to be effective as a last line of defense for reducing fumonisin exposure. Biologically based methods for control of fumonisin-producing *Fusarium* spp. and decontamination of the fumonisins could have potential commercial application, while simple and practical intervention strategies could also impact positively on food safety and security, especially in rural populations reliant on maize as a dietary staple.

## Introduction

***Fusarium*** spp. are agriculturally important **plant pathogenic fungi** associated with disease and **mycotoxin** contamination of grain crops (Wild and Hall, 2000; Picot et al., 2011). *Fusarium* ear rot in maize is one of the major diseases affecting maize production worldwide and poses an enormous threat to the international trade of foods and feeds. Fungal species of *Fusarium* Section Liseola, including *Fusarium verticillioides, Fusarium proliferatum*, and *Fusarium subglutinans* are some of the most important causative fungal agents of *Fusarium* ear or kernel rot as well as symptomless infection of maize crops, leading to contamination with the **fumonisin** mycotoxins (Munkvold et al., 1997).

Fifteen *Fusarium* spp. have been reported to produce fumonisins. Eight species are from the Section Liseola, i.e., *F. verticilloides, Fusarium sacchari, Fusarium fujikuroi, F. proliferatum, F. subglutinans, Fusarium thapsinum, Fusarium anthophilum*, and *Fusarium globosum* (Rheeder et al., 2002). Another five species fall within Section Dlaminia, i.e., *Fusarium nygamai, Fusarium dlamini*, and *Fusarium napiforme*. Trace amounts of fumonisin were detected in culture material of two species, i.e., *Fusarium andiyazi* and *Fusarium pseudonygamai*. The remaining two fumonisin-producing *Fusarium* spp. are one species in Section Elegans, i.e., *Fusarium oxysporum* and one in Section Arthrosporiella, i.e., *Fusarium polyphialidicum*. The fumonisins are associated with several diseases in humans, animals, poultry, and fish (Marasas, 2001; Marasas et al., 2004; Kimanya et al., 2010) and are classified as Group 2B carcinogens (IARC, 2002). Home-grown maize is a major dietary staple in southern Africa and known to be frequently contaminated with unacceptable levels of fumonisins, with fumonisin B_1_ (FB_1_) being the most prevalent natural occurring fumonisin (Marasas, 2001; Marasas et al., 2004; Shephard et al., 2007, 2013; Burger et al., 2010). The Eastern Cape Province of South Africa is one of the areas in the world where the highest levels of FB_1_ were recorded in home-grown maize. As a result exposure to FB_1_ in adults is more than four times above the provisional maximum tolerable daily intake (2 μg FB_1_/kg body weight/day) set by the Joint Food and Agriculture Organization of the United Nations and the World Health Organization (FAO/WHO) Expert Committee on Food Additives (Bolger et al., 2001).

The fumonisins comprise a group of 28 characterized analogs, which can be separated into four main groups: fumonisin A, B, C, and P (Rheeder et al., 2002). The fumonisin B (FB) analogs, which includes FB_1_, FB_2_, and FB_3_, are the most abundant naturally occurring fumonisins, with FB_1_ predominating and usually being found at the highest levels. Apart from FB, some of the other analogs may occur in naturally contaminated maize at relatively low levels. The complete fumonisin molecule plays an important role in toxic and cancer-initiating activities *in vivo* (Gelderblom et al., 1993). Studies evaluating the structure-activity relationship of fumonisin analogs, hydrolysis products and a monomethyl ester of FB_1_ in short-term carcinogenesis in rats and cytotoxicity assays in primary rat hepatocytes, indicated that the free amino group plays a pivotal role in the toxicological effects of the fumonsins *in vitro* and *in vivo*. It was suggested that the tricarballylic acid moiety is required for effective absorption of the fumonisins from the gut. The fumonisins disrupt sphingolipid biosynthesis by inhibiting the enzyme ceramide synthase (Wang et al., 1991), and the tricarballylic acid moiety is required for maximal effect (Van der Westhuizen et al., 1998).

*Fusarium* infect maize in the field with the highest levels of fumonisins present at harvest, concentrated in the pericarp and embryo of the maize kernel (Fandohan et al., 2006; Kimanya et al., 2008; Burger et al., 2013). Kinetics of *Fusarium* growth and mycotoxin production are mainly affected by water activity, temperature, and atmospheric composition, while nutritional factors such as kernel endosperm composition and nitrogen sources also play an important role (Chulze, 2010; Picot et al., 2011). Fumonisin production strongly depends on the kernel stage, and may be regulated by physicochemical factors that vary during ear ripening. Insect damage of maize by the European corn borer (*Ostrinia nubilalis* Hübner) and the corn earworm (*Helicoverpa zea* Boddie) further favors *Fusarium* infection (Betz et al., 2000).

**Methods for reduction** of fumonisins in maize are applied pre-harvest or during harvesting and processing (Wild and Gong, 2010). These include several existing strategies to reduce *Fusarium* growth and production of fumonisins in food sources, i.e., controlled agricultural practices, ensiling strategies, breeding for insect and fungal resistance in maize cultivars, various physical-, chemical-, and biological treatment methods and genetic engineering approaches. Good agricultural management and hazard analysis and critical control point (HACCP) practices promote the general condition of crops, reducing but not eliminating fungal growth, and mycotoxin contamination, while resistance breeding strives to achieve a balance between developing resistant crops and maintaining high quality crop yield (Cleveland et al., 2003; Wild and Gong, 2010). However, optimization of agricultural management practices is not always possible due to high production costs, the geographical location or nature of the production systems, and challenging environmental conditions.

Several physical and chemical control methods for mycotoxins have been commercialized involving sorting and flotation, solvent extraction, chemical detoxification by alkalization (e.g., ammonia, sodium hydroxide, and sulfur dioxide treatments), oxidation (e.g., ozone), and irradiation and pyrolysis (He and Zhou, 2010). There are, however, several limitations, challenges, and concerns with regards to physical and chemical control methods (Schatzmayr et al., 2006). Physical methods generally have low efficacy and less specificity, while chemical methods are not always effective, are considered expensive and may decrease the nutritional value of foods, affect the sensory quality, and could produce toxic derivatives (Alabouvette et al., 2009; He and Zhou, 2010). Furthermore, methods involving fungicides pose a potential health, safety, and environmental risk as certain antifungal chemical compounds are not biodegradable or have a long degradation period, could contaminate soil and water and their effect on food quality and human health is a concern (Larkin and Fravel, 1998; da Cruz Cabral et al., 2013). Prolonged chemical treatment of grains can lead to the development of resistance in fungal strains, a demand for higher concentrations, and an increase in toxic residues in food crops. Increasingly more stringent regulation is enforced with regards to the use of chemical control methods together with a strong consumer demand to reduce the use of potentially harmful chemicals in the food supply (Liu et al., 2013). There is also an ecological and societal movement toward safe and natural food, without chemical treatments and/or preservatives (Edlayne et al., 2009).

Research over the past 25 years indicates support for agricultural management practices and a renewed interest in **practical and biological control methods** as possible alternatives. In this regard several methods for controlling fungal growth and mycotoxin production pre- and post-harvest involving clay minerals, plant extracts and a variety of microbial taxa have been commercialized (He and Zhou, 2010). In rural **subsistence farming communities** a number of effective, practical, and culturally acceptable intervention methods have been developed (Kimanya et al., 2008; Van der Westhuizen et al., 2010). While the focus in the past was more on the most economically important mycotoxins, i.e., aflatoxin B_1_ (AFB_1_), much less information is available on other important mycotoxins such as FB_1_, trichothecenes, zearalenone, citrinin, and patulin (Kabak et al., 2006). This paper presents a comprehensive overview of recent research on biological- and practical-based approaches for control of fumonisin-producing *Fusarium* spp. and **methods for reduction** thereof during pre- and post-harvest conditions. Current information on the application of natural clay adsorbents, biocontrol organisms, antioxidants, essential oils, plant extracts, and molecular approaches are reviewed; as well as practical and culturally acceptable methods for reduction of fumonisin exposure in rural subsistence farming communities.

## Pre-harvest biologically based control methods for fumonisin-producing *Fusarium* spp.

### Biocontrol microorganisms

This approach involves a three-way interaction between the host commodity, the pathogen and the antagonistic biocontrol microorganism together with dynamics such as competition for nutrients and space, parasitism of the pathogen, secretion of antifungal compounds, induction of systemic resistance (ISR), biofilm formation and involvement with reactive oxygen species in defense response (Larkin and Fravel, 1998; Alabouvette et al., 2009). Recent research also suggested that the aflatoxin biocontrol mechanism, employing atoxigenic strains of *Aspergillus flavus*, is triggered by physical contact or interaction between hyphae of the competing fungal strains (Damann, 2014). Essential criteria for effective biocontrol microorganisms include the ability to colonize the plant part infected by the pathogen organism, efficacy under the relevant environmental conditions and compatibility with other control methods that are applied (Bacon and Hinton, 2011; Liu et al., 2013). Niche overlap indices (NOIs) provide information on ecological similarity, coexistence, and competition between microorganisms in a specific niche and assists in identifying possible microbial antagonists against *F. verticillioides* colonization (Cavaglieri et al., 2004). Microorganisms naturally associated with and adapted to the vegetative parts of a specific plant, sharing the ecological niche with pathogen microorganisms, could hold advantages as biocontrol agents. One such a microorganism, *Bacillus subtilis* occupies the same ecological niche as *F. verticillioides* within the maize plant and effectively inhibits growth of the fungus, based on competitive exclusion (Bacon et al., 2001; Table 1). *B. subtilis* is considered generally regarded as safe (GRAS) by the United States Food and Drug Administration [US FDA, GRAS substances evaluated by the Select Committee on GRAS substances (SCOGS)], is easy to cultivate and manipulate genetically, and therefore suitable for industrial processes. A pre-harvest biological control system, involving *B. subtilis* RRC101, was developed on maize which reduces fumonisin accumulation during the endophytic growth phase of *F. verticillioides* (= *F. moniliforme*; Bacon et al., 2001). The endophytic phase of *F. verticillioides* is transferred vertically to the next generation through clonal infection of seeds. This phase is characterized by intercellular systemic infection of plants and seeds, which cannot be controlled with fungicides. Effective biocontrol has also been demonstrated with wild type and fusaric acid resistant mutant strains of the bacterial endophyte, *Bacillus mojavensis, in vitro* and *in planta* (Bacon and Hinton, 2011). Efficacy of these strains under field conditions could be influenced by fusaric acid produced by *F. verticillioides*. The mechanism of biocontrol by *B. mojavensis* is complex and still unclear, as indicated by broad differences in maize seedling protection by a range of strains evaluated.

**Table 1:** **Current information on reduction of fumonisin-producing ***Fusarium*** spp. by biocontrol microorganisms ***in vitro, in planta***, and in field trials**.

**Biocontrol microorganism**	***Fusarium* spp. studied**	**Test system with details of experimental model**	**Reduction criteria**	**Application**	**Reference(s)**
*Trichoderma* spp. strains aggressive toward *Fusarium verticillioides* (*= moniliforme*): *Trichoderma harzianum* T1 and T2, *Trichoderma viride* T5 and T6	*F. verticillioides* (*= F. moniliforme*)	*In vitro* studies on the potential for biological control of *Aspergillus flavus* and *F. moniliforme* by *Trichoderma* spp.: a study of the production of extracellular metabolites by *Trichoderma* spp. *In vitro:* Effect of carbon source on antifungal properties of *Trichoderma* spp.; Antifungal activities of *Trichoderma* spp. culture filtrates: preparation of liquid culture filtrates; PDA plate assay; determination of inhibition; scanning electron microscopy of mycelial plugs; Production of volatiles: inverted fungal cultures; colony diameters; Production of extracellular enzymes: agar plate method; measurement of depletion of nutrient sources; Evaluation of osmotic potential; Production of antibiotics: solid agar plate assay; monitoring zones of inhibition of *E. coli* and *Staphylococcus aureus*	*In vitro:* Effect of carbon source on antifungal properties of *Trichoderma* spp.: *Trichoderma* spp. inhibited *F. verticillioides* growth on growth medium with glucose as carbon source; no inhibition with sucrose as carbon source; inhibition observed with L-alanine as nitrogen source; *T. harzianum* T2 and *T. viride* T5 exhibited the strongest inhibitory effect; Antifungal activities of *Trichoderma* sp. culture filtrates: general inhibition of *F. verticillioides* growth; culture filtrates of *T. harzianum* T2 and *T. viride* T5 resulted in pronounced morphological alterations; Production of volatiles: the presence of volatile compounds of *T. harzianum* T2, *T. viride* T5 and T6 were able to suppress *F. verticillioides* growth; Production of extracellular enzymes: amylase and cellulose activity exhibited by all four strains; lypolytic activity exhibited by *T. harzianum* T1, T2 and *T. viride* T5; proteolytic activity exhibited by *T. harzianum* T2 and *T. viride* T5; extracellular pectinolytic activity exhibited by *T. harzianum* T1 and *T. viride* T5. *T. viride* produced the widest spectrum of extracellular enzymes; Evaluation of osmotic potential: enzyme production decreased with increasing osmotic potential; Production of antibiotics: *T. viride* T5 exhibited the greatest inhibitory effect on *E. coli* and *S. aureus*, suggesting production of antibiotics	*Trichoderma* spp. exhibited potential for biocontrol against mycotoxin-producing fungi; the lack of osmotolerance in air-dried seed could be a disadvantage	Calistru et al., 1997
*T. viride* UPS101 isolated from root segments of corn plants grown in Piedmont, Georgia, USA	*F. verticillioides* (*= F. moniliforme*) strains: RRCPAT, RRCPATgus	*T. viride* suppresses FB_1_ production by *F. moniliforme*. *In vitro:* Antifungal activity: single and co-cultivation on PDA; colony diameters; Effect on FB_1_ levels: single and co-cultivation on maize kernels; determination of FB_1_ levels	*In vitro:* Antifungal activity: *T. viride* suppressed radial extension of *F. verticillioides* colonies (46% reduction after 6 days; 90% after 14 days); Effect on FB_1_ levels: *T. viride* suppressed FB_1_ production by *F. verticillioides* when co-cultivated on maize kernels; 85% reduction in FB_1_ levels when the *T. viride* and *F. verticillioides* were inoculated simultaneously; 72% reduction in FB_1_ levels when *T. viride* was inoculated 7 days after *F. verticillioides*	*Trichoderma* spp. mainly applied to soil as biocontrol agents; *T. viride* could be applied to inhibit *F. verticillioides* growth pre-harvest, to prevent disease during plant development, postharvest during storage or to suppress FB_1_ accumulation in inadequately dried maize kernels; applicable for FB_1_ reduction in maize kernels intended for animal feed	Yates et al., 1999
*Bacillus subtilis* strains isolated from maize in northern Italy: *B. subtilis* RRC101 (wild type) (Patent 5, 994, 117), B. *subtilis* RRC26ss and *B. subtilis* RRC24wf (rifampicin resistant mutants);	*F. verticillioides (= moniliforme)* Wild type strains: MRC826, RRC410, RRCPAT, RRC408; *F. verticillioides* transformed ecological marker strains: MRC826gus, RRCPATgus, RRC408gus	Biological control of *F. moniliforme* in maize. *In planta*: Young and vigorous maize seedlings: Plant pot cultures subjected to drought treatments; seed treated with *B. subtilis*; plants cultivated in soil infested with *F. verticillioides*; plant growth light room; determination of seedling height and blade width; percentage seedling root infection; CFU counts of *B. subtilis* and *F. verticillioides* in soil; determination of FB_1_ levels; Mature maize plants: Ten week old maize plants; determination of FB_1_ levels in roots, stems, leaves and kernels	*In planta*: Young and vigorous maize seedlings: *B. subtilis* exhibited a protective effect on maize seedling growth and percentage seedling root infection; *B. subtilis* reduced *F. verticillioides* colonization of soils; FB_1_ was significantly reduced (50%) by all bacterial treatments, especially under drought stress; Mature maize plants: *B. subtilis* exhibited protection in matured plants at the kernel fill stage	*B. subtilis* could be applied as seed treatment to act as biocontrol agent during the growth of maize plants; evaluation of *B. subtilis* under field conditions needed	Bacon et al., 2001
A large variety of potential antagonistic bacterial and fungal strains isolated from straw, stubble, seed surfaces, and the phylosphere or roots of cereal crops; Additional isolates: *Chaetomium* spp., *Fusarium equiseti*	*Fusarium* isolates from infected wheat grains in The Netherlands: *Fusarium culmorum, Fusarium graminearum, Fusarium proliferatum, F. verticillioides*	Potential of fungal antagonists for bio-control of *Fusarium* spp. in wheat and maize through competition in crop debris. *In vitro:* Reduction of *Fusarium* spp. conidia formation: wheat straw bioassay; pre-inoculation of straw with *Fusarium* spp.; subsequent inoculation of straw with potential antagonists; determination of the number of conidia: microscopy bioassay; Reduction of *Fusarium* spp. conidia formation: maize stubble bioassay; procedures similar to the wheat straw bioassay; Field trials: Maize stalks: determination of antagonism; pre-inoculation of stalks with potential antagonists; subsequent inoculation of stalks with *F. verticillioides, F. proliferatum* and *F. graminearum*; plots inoculated with strips containing stalk pieces; culturing of harvested stalks on modified PDA; identification of *F. verticillioides, F. proliferatum* and *F. graminearum*: colony morphology and microscopic examination; Maize ears: pre-inoculation of silks with potential antagonists; silk of tagged ears at the blooming stage; subsequent inoculation of silks with *F. verticillioides, F. proliferatum* and *F. graminearum;* identification of *F. verticillioides, F. proliferatum* and *F. graminearum*: colony morphology and microscopic examination	*In vitro*: Reduction of *Fusarium* spp. conidia formation (wheat straw bioassay): sporulation of *F. culmorum* and *F. graminearum* on straw: overall reduction (>80%) by antagonistic isolates; Sporulation of *F. culmorum* on *Clonostachys rosea*-treated straw: 85-99% reduction; sporulation of *F. graminearum* on *C. rosea*-treated straw: 91-100% reduction; Highly effective fungal antagonists: *C. rosea, F. equiseti, Chaetomium globosum* and *Epicoccus nigrum;* non-pathogenic *Fusarium* spp. exhibited moderate antagonism; Yeasts were weak competitors; Reduction of *Fusarium* spp. conidia formation (maize stubble bioassay): less effective reduction in sporulation than reported for wheat straw; strongest antagonist: *C. rosea*; Field trials: Maize stalks: Variation in results; Most consistent reduction of *Fusarium* colonization by *C. rosea*; Maize ears: Ear treatments with *C. rosea* and *Cladosporium cladosporioides* reduced colonization of kernels with both *F. verticillioides* and *F. graminearum* (50% reduction); *F. proliferatum* colonization reduced by *C. cladosporioides* and *F. equiseti*	Application of antagonists on flowering maize ears: promising results in preliminary field trials; further experiments under disease conducive conditions needed; several antagonists exhibited potential to control *Fusarium* spp. in wheat and maize crop residues postharvest, and at the flowering ear stages	Luongo et al., 2005
Lactic acid bacterial isolates from maize tissues collected in maize fields (66 isolates); *Pediococcus pentosaceus* L006	A variety of *F. verticillioides* and *F. proliferatum* strains from the INRA MycSA collection	Potential of *P. pentosaceus* (L006) isolated from maize leaf to suppress fumonisin-producing fungal growth. *In vitro:* Antifungal activity against *F. verticillioides* and *F. proliferatum:* Overlay MRS agar plate method; selection of the most efficient isolate; Identification of the most efficient antifungal lactic acid bacterial isolate: biochemical and physiological characterization (API 50 CHL test); 16S rRNA gene sequencing; Antifungal spectrum of *P. pentosaceus* L006 on solid medium: *P. pentosaceus* L006 tested against a range of *F. verticillioides* and *F. proliferatum* strains; Overlay MRS agar plate method; Production of active antifungal metabolites by *P. pentosaceus* L006: cultivation in MRS broth; sampling during 120 h incubation period; measurement of pH, cell growth and antifungal activity of cell-free supernatant; determination of antifungal activity; Characterization of *P. pentosaceus* L006 cell-free culture supernatant: determination of heat stability and the effects of pH and proteolytic enzyme treatments on antifungal activity	*In vitro*: Antifungal activity against *F. verticillioides* and *F. proliferatum:* 89% of lactic acid bacterial isolates were able to inhibit fungal growth; antifungal activity maximal toward the end of the exponential phase of growth; Identification of the most efficient antifungal lactic acid bacterial isolate: *P. pentosaceus* L006 (100% sequence similarity with *P. pentosaceus* ATCC 25745); Antifungal spectrum of *P. pentosaceus* L006 on solid medium: *P. pentosaceus* L006 inhibited the growth of all fungal strains tested; Production of active antifungal metabolites by *P. pentosaceus* L006: antifungal activity increased with incubation time; antifungal substances are possibly secondary metabolites; pH decreased (pH 6.5 to 3.8) during incubation; Characterization of *P. pentosaceus* L006 cell-free culture supernatant: antifungal activity was not reduced by heat and proteolytic enzyme treatments; antifungal compounds not proteinaceous; antifungal activity lost at pH 7; antifungal activity was ascribed to the presence of organic acids, excluding lactic acid	Application of *P. pentosaceus* L006 can possibly improve silage quality; results obtained *in vitro* need to be extended to *in planta* studies and field trials	Dalie et al., 2010
*Bacillus mojavensis* strains RCC101 (ATCC 55732) (patented); NRRL B14699; NRRL B14701; NRRL B14703 to NRRL B14706; NRRL B14708 to NRRL B14712; *B. mojavensis* rifampin mutant RRC112rif; *B. mojavensis* fusaric acid resistant mutant RRC112fa	*F. verticillioides* strains: MRC 826 (symptomless endophytic strain), “Patgus” (virulent strain), 408 (virulent wild type strain), and UV28 (non-fusaric acid producing mutant strain)	*In planta* reduction of maize seedling stalk lesions by the bacterial endophyte *B. mojavensis*. *In planta:* *B. mojavensis* (strains RCC101; NRRL B14699; NRRL B14701; NRRL B14703 to NRRL B14706; NRRL B14708 to NRRL B14712) inoculated *Zea mays* “Early Sunglow” seeds were cultivated for 35 days in a plant growth light room and inoculated with a spore suspension of *F. verticillioides* “Patgus”; *B. mojavensis* RRC112fa inoculated *Zea mays* “Pioneer 3140” seeds were cultivated and inoculated as described above with *F. verticillioides* strains MRC 826, 408 and UV28; *B. mojavensis* RRC112fa inoculated *Zea mays* “Early Sunglow” seeds were cultivated as described above and inoculated with *F. verticillioides* strains “Patgus” and UV28; Determination of stalk lesion development; Measurement of the length of necrotic lesions and stalk diameters	*In planta:* Range of *B. mojavensis* strains + *F. verticilloides* “Patgus” (“Early Sunglow” maize): 24-58% reduction in stalk lesion length; large differences in the ability to reduce lesions; *B. mojavensis* RCC101 exhibited 58% reduction; *B. mojavensis* RRC112fa + *F. verticillioides* strains MRC 826, 408 and UV28 (“Pioneer 3140” maize): 30-41% reduction in stalk lesion length; *B. mojavensis* RRC112fa significantly (*P* = 0.05) reduced stalk lesion lengths caused by *F. verticillioides* “Patgus” on “Early Sunglow” maize (54% reduction); *B. mojavensis* RRC112fa: 70% reduction in stalk lesion length; reduction not significantly different from results for the RRC101 wild type strain and rifampin mutant strain (RRC112rif); Significant (*P* ≤ 0.05) reduction in stalk lesion length by the bacterium regardless of its ability to tolerate fusaric acid; *F. verticilloides* UV28 significantly (*P* ≤ 0.05) reduced maize stalk diameter; no enhanced effect when the fungus was co-inoculated with *B. mojavensis*	Application of *B. mojavensis* for suppression of seedling disease in maize: to proof the efficacy of *B. mojavensis* as biocontrol agent additional studies should be performed *in vitro* and in the field utilizing mutants and wild-type strains of bacteria and non-fumonisin producing fungi; more pathological factors should also be evaluated	Bacon and Hinton, 2011

*FB_1_, Fumonisin B_1_; CFU, Colony forming units; PDA, Potato dextrose agar; MRS broth/agar, de Man, Rogosa and Sharpe broth/agar*.

*Pediococcus pentosaceus*, a lactic acid bacterial isolate from maize, inhibits *F. verticillioides* and *F. proliferatum* growth *in vitro* (Dalie et al., 2010; Table 1). Antifungal activity in *P. pentosaceus* culture supernatant was observed toward the end of the exponential phase of growth and was pH dependent. The antifungal metabolites produced proved to be heat stable and resistant to proteolytic enzymes. Culture fractions exhibiting antifungal activity contained compounds with molecular masses ranging from 500 to 1400 Da. *P. pentosaceus* has GRAS status, has been widely used in the fermentation of a variety of foods and could be suitable as biocontrol organism to improve the quality of ensilage. *Clonostachys rosae*, a fungal isolate from straw, stubble, seed surfaces, and the phylosphere or roots of cereal crops, effectively reduced sporulation of *F. verticillioides* and *F. proliferatum* on maize stalks *in vitro* and in field trials (Luongo et al., 2005). *C. rosae* exhibited potential to control *Fusarium* spp. in maize at the flowering ear stages and in crop residues post-harvest. Food-grade yeasts are also considered ideal biocontrol microorganisms, as they are generally genetically stable, effective at low concentrations, easy to cultivate, capable to survive under adverse environmental conditions, compatible with commercial processing, and resistant to pesticides.

### *Trichoderma* spp.

*Trichoderma* spp. are considered effective biocontrol agents because of their repertoire of extracellular lytic enzymes that cause necrotrophic action through lysis of fungal cell walls as well as the role they play in ISR in plants (Bacon et al., 2001; Hermosa et al., 2012). *Trichoderma* mainly colonizes the rhizosphere and intercellular root areas of plants, and maintains interactions by promoting plant growth and providing protection against infections, while utilizing plant sucrose to facilitate root colonization (Hermosa et al., 2012). Plant disease severity is reduced in the presence of *Trichoderma* by inhibition of a wide range of plant pathogens through antagonistic and mycoparasitic action; ISR or induction of localized resistance. *Trichoderma* is also able to withstand toxic metabolites that are produced by the plant in response to invasion. Plants are able to detect pathogen- or microbe associated molecular patterns (MAMPs), which leads to activation of defense mechanisms and eventually synthesis of antimicrobial compounds. Certain *Trichoderma* strains produce a variety of MAMPs, contributing to activation of plant defense responses. Salicylic acid, jasponic acid and ethylene play a key role in plant immunity and hormone-signaling pathways as well as defense response pathways of the hormones abscisic acid, indole-3-acetic acid, and gibberellin (Pieterse et al., 2009). Indole-3-acetic acid produced by *Trichoderma* contributes to ethylene biosynthesis, which in turn stimulates abscisic acid biosynthesis. Depending on *Trichoderma* stimuli, phytohormone homeostasis will control plant development and immune responses. *Trichoderma* chitinases also release fungal chitin oligosaccharides, and elicit ISR by jasmonic acid/ethylene dependent pathways, thereby triggering defense responses in plants. A polyketide synthase/non-ribosomal peptide synthetase hybrid enzyme of *Trichoderma virens* is involved in plant interactions and was shown to induce plant defense responses (Mukherjee et al., 2012). Several *Trichoderma* spp. with GRAS status, including *Trichoderma viride* and *Trichoderma harzianum*, are capable of effectively reducing *F. verticillioides* (= *F. moniliforme*) growth and fumonisin production *in vitro* and *in planta* (Calistru et al., 1997; Larkin and Fravel, 1998; Yates et al., 1999; Table 1). The inhibitory effect on *F. verticillioides* growth when co-cultured with *Trichoderma* spp. can be attributed to antibiosis through production of volatile compounds, extracellular enzymes and antibiotics. The antagonistic fungal species *T. viride* is widely used in bio-fertilizers for biological control of soil borne plant-pathogenic fungi in crops.

### Non-pathogenic biocontrol strains

Non-pathogenic strains of pathogenic species are often applied for biocontrol (Liu et al., 2013). In this regard, moderate suppression of toxigenic *F. verticillioides* and *F. proliferatum* strains by non-pathogenic *Fusarium* strains was demonstrated by Luongo et al. (2005; Table 1).

The development of *Fusarium* biocontrol strains with reduced mycotoxin production ability through RNA silencing technology may be a useful tool for reducing mycotoxin contamination in agricultural products (McDonald et al., 2005). Transformation of *F. graminearum* with inverted repeat transgenes (IRT) containing sequences of mycotoxin-specific regulatory genes results in suppression of mycotoxin production. Other gene silencing techniques involving deletion of *ZFR1* of *F. verticillioides*, which regulates sugar transporter genes and in turn affect fumonisin biosynthesis during kernel colonization, resulted in significantly less growth on maize kernel endosperm tissue (Bluhm et al., 2008).

### Rhizobacteria

*Fusarium verticillioides* is the most prevalent *Fusarium* spp. present in the rhizoplane and endorhizosphere areas of maize, while *Arthrobacteria* and *Azotobacter* are the predominant bacterial genera (Cavaglieri et al., 2005a). Pathogens germinate and colonize roots within a few days of planting, while biocontrol rhizobacteria could be metabolically active during this period. A number of rhizobacterial isolates of maize plants sampled from a commercial maize field and exhibiting high NOIs with *F. verticillioides*, including *Arthrobacter globiformis, Azotobacter armeniacus, Pseudomonas solanacearum, B. subtilis, Enterobacter cloacae*, and *Microbacterium eoleovorans* exhibited antifungal activity *in vitro* by effectively reducing *F. verticillioides* growth and FB_1_ production on maize meal extract agar (Cavaglieri et al., 2004, 2005a,b,c) (Table 2). Maize seeds pre-treated with *A. armeniacus* RC2, A. *globiformis* RC5, *E. cloacae, M. eoleovorans*, and *Bacillus* sp. CE1 and evaluated *in planta*, resulted in effective reduction of *F. verticillioides* growth in the rhizoplane and endorhizosphere areas. A good correlation was observed between results obtained from *in vitro* and *in planta* studies (Cavaglieri et al., 2005c). *Enterobacter cloacae* exhibited potential for biocontrol of root colonization by *F. verticillioides*. Inducible Type 1 fimbrae of *E. cloacae* may play a role in the colonization of roots (Hinton and Bacon, 1995). Rhizobacterial strains could have potential application as seed inoculants to reduce *F. verticillioides* colonization on root level, in the rhizoplane and endorhizosphere areas (Cavaglieri et al., 2005c). Effectiveness of a biocontrol organism to colonize the rhizosphere and its value as biocontrol agent could, however, be influenced by environmental conditions and the initial cell concentrations of the biocontrol organism and the pathogen.

**Table 2:** **Current information on reduction of ***Fusarium verticillioides*** growth and fumonisin B_**1**_ production by rhizobacteria ***in vitro*** and ***in planta*****.

**Rhizobacterial microorganism**	***Fusarium* sp. studied**	**Water activity (a_*w*_)**	**Test system with details of experimental model**	**Reduction criteria**	**Application**	**Reference(s)**
Rhizobacterial isolates from maize plants in Italy: *Enterobacter cloacae*	*F. verticillioides* (= *F. moniliforme*): Isolates from maize: MRC 826, RRC 374, RRC 408, Isolates from rice: RRC 410	N/A	*E. cloacae* is an endophytic symbiont of corn. Reduction of *F. verticillioides* root colonization of maize seedlings by *E. cloacae:* *In planta*: Distribution of *E. cloacae* root colonization: sterile maize seed inoculated with *E. cloacae* and cultured in tubes with soil; cultured in plant growth rooms under light; microscopic examination of root colonization; *In vitro*: Cultivation of inoculated seeds on PDA, damp filter paper or sterile soil; microscopic examination of root colonization; Determination of antagonism: co-cultivation on PDA; examination of zones of inhibition; microscopic examination of seedling roots: light microscopy; transmission electron microscopy; scanning electron microscopy	*In planta*: *E. cloacae* root colonization: *E. cloacae* biologically associated with maize seedling roots; observed internally and in the rhizoplane areas; on maize seedlings *E. cloacae* was distributed over the epidermis and internally in several locations of the cortex; no bacteria observed in the endodermis, but intercellular within the outer margin of the pericycle; *E. cloacae* not observed in the pith area; present in stems and leaves; *E. cloacae* distributed externally along the secondary and primary seedling roots as well as the root cap of the primary root; a matrix-like capsule observed surrounding the bacterial cells on the external surface of the primary root; *E. cloacae*: no damage to host cells; no reduction in percentage germination or time of germination; Determination of antagonism: all bacterial isolates inhibited growth of *F. verticillioides* strains	*E. cloacae* exhibited potential for biocontrol of root colonization by *F. verticillioides;* the endophytic association of *E. cloacae* with maize enhances its potential as biocontrol agent	Hinton and Bacon, 1995
Rhizobacterial isolates from maize roots, sampled from a commercial maize field: *Arthrobacter globiformis, Azotobacter armeniacus, Pseudomonas solanacearum, B. subtilis*	*F. verticillioides* isolates from maize roots sampled from a commercial maize field	0.937; 0.955; 0.982	Screening procedures for selecting rhizobacterial strains with biocontrol effects upon *F. verticillioides* growth and FB_1_ production: *In vitro*: Determination of NOIs: utilization of 17 compounds in maize as sole carbon source; selection of isolates with the highest NOIs; Antibiosis and antifungal activity of selected isolates: 2% MMEA; adjustment of a_w_ levels; inoculation and incubation; measurement of zones of inhibition and colony diameters; FB_1_ levels in MMEA cultures: HPLC analyses	*In vitro*: Determination of NOIs: information on ecological similarity and coexistence with *F. verticillioides;* percentage isolates able to utilize all carbon sources: a_w_ 0.937 (58%), 0.955 (20%) 0.982 (75%); most competent strains: *Arthrobacter* strains at all a_w_ levels; *A. armeniacus, P. solanacearum* and *B. subtilis* competent at a_w_ 0.955 and 0.937; Antibiosis and antifungal activity of selected isolates: all bacterial isolates effectively inhibited *F. verticillioides* growth; most effective growth inhibition (*P* < 0.001): *A. globiformis* and *B. subtilis* isolates; all isolates significantly (*P* < 0.001) reduced the growth rate and increased the lag phase of fungal growth; *B. subtilis* strains exhibited the strongest effects; FB_1_ levels in MMEA cultures: reduced FB_1_ levels exhibited at all a_w_ levels evaluated; *P. solanacearum* and *B. subtilis:* 70-100% reduction at all a_w_ levels; *A. armeniacus:* 65% reduction at a_w_ 0.955	*A. armeniacus* RC2 and RC3; *B. subtilis* RC8, RC9 and RC11; *P. solanacearum* RC7 and RC10 could have value for control of *F. verticilloides* root colonization	Cavaglieri et al., 2004
Predominant bacterial isolates colonizing the maize endorhizosphere and isolated from maize roots, sampled from a commercial maize field: *A. globiformis*, *A. armeniacus*	Toxigenic *F. verticillioides* maize endorhizosphere isolates from maize roots, sampled from a commercial maize field	0.937; 0.955; 0.982	Rhizobacteria and their potential to control *F. verticillioides*: effect of maize bacterization and inoculum density: *In vitro*: *F. verticillioides* isolates paired with each bacterial strain in dual culture; Antibiosis and effect on fungal growth rate: MMEA; inoculation and incubation; measurement of zones of inhibition; measurement of colony diameters; FB_1_ levels in MMEA cultures: HPLC analyses Greenhouse studies: Effect of separate and combined bacterial treatments on *F. verticillioides* root colonization in the rhizoplane and endorhizosphere areas; inoculation of seeds with rhizobacterial strains; modified tube assay; determination of *F. verticillioides* CFU counts in the rhizoplane and endorhizosphere areas	*In vitro:* Antibiosis and effect on fungal growth rate: effective inhibition of fungal growth at a_w_ 0.955 and 0.982; *A. armeniacus* RC2 and RC3 inhibited fungal growth of 60-100% *F. verticilliodes* strains at a_w_ 0.955–0.982; *A. globiformis* RC4 and RC5 inhibited fungal growth of 69-80% of *F. verticilliodes strains* at a_w_ 0.955-0.982; *A. armeniacus* RC2 reduced (56-75%) FB_1_ accumulation at a_w_ 0.955; *A. globiformis* RC4 and RC5 reduced (20-96%) FB_1_ accumulation at a_w_ 0.955 and 0.982; Greenhouse studies: Seeds treated with *A. armeniacus* RC2 and *A. globiformis* RC5: 100% inhibition of fungal growth in the rhizoplane and endorhizosphere areas; bacterial mixture treatment resulted in 100% inhibition of fungal growth in the endorhizosphere area	*A. armeniacus* RC2 exhibited potential as maize seed inoculant for reduction of *F. verticillioides* root colonization	Cavaglieri et al., 2005a
Endorhizosphere bacterial isolates from maize roots, sampled from a commercial maize field: Bacterial mixture 1: *E. cloacae*, *Microbacterium eoleovorans* Bacterial mixture 2: *P. solanacearum, B. subtilis*	Toxigenic *F. verticillioides* maize endorhizosphere isolates from maize roots, sampled from a commercial maize field	0.937; 0.955; 0.982	*In vitro* influence of bacterial mixtures on *F. verticillioides* growth and FB_1_ production: effect of seeds treatment on maize root colonization: *In vitro:* Antibiosis: MMEA; adjustment of a_w_ levels; *F. verticillioides* isolates paired with each bacterial mixture in dual culture; different bacterial inoculum sizes evaluated (10^8^, 10^9^ and 10^10^ cells/ml); measurement of zones of inhibition; Antifungal activity: MMEA; adjustment of a_w_ levels; pour-plate method; inoculation with *F. verticillioides* isolates; measurement of colony diameters; FB_1_ levels in MMEA cultures: HPLC analyses; Greenhouse studies: Effect of combined bacterial seed treatments on *F. verticillioides* root colonization in the rhizoplane and endorhizosphere areas; inoculation of seeds with bacterial mixtures; determination of *F. verticillioides* CFU counts in the rhizoplane and endorhizosphere areas	*In vitro:* Bacterial mixture 1: Antibiosis: fungal growth significantly (*P* < 0.05) reduced at all a_w_ levels and inoculum sizes; inoculum size 10^8^ cells/ml exhibited the strongest effect; Antifungal activity: significant (*P* < 0.05) decrease in fungal growth rate at a_w_ 0.955 and 0.937 with all inoculum sizes; reduction in fungal growth rate obtained at a_w_ 0.982 with 10^9^ and 10^10^ cells/ml; FB_1_ production: only reduced at a_w_ 0.955 by all inoculum sizes; Bacterial mixture 2: Antibiosis: fungal growth most effectively (*P* < 0.05) reduced at a_w_ 0.937 with 10^8^ and 10^9^ cells/ml; Antifungal activity: significant (*P* < 0.05) reduction in fungal growth rate obtained at a_w_ 0.982 with 10^10^ cells/ml and at a_w_ 0.955 with 10^9^ cells/ml; no effect with 10^8^ cells/ml; FB_1_ production not reduced by any of the inoculum sizes; *In planta*: Bacterial mixture 1 (10^8^ cells/ml) completely inhibited *F. verticillioides* growth; Bacterial mixture 2: inhibition of *F. verticillioides* growth not consistent	*E. cloacae* and *M. oleovorans* exhibited potential as maize seed inoculants for reduction of *F. verticillioides* root colonization, i.e. prevention of vertical transmission of *F. verticillioides*	Cavaglieri et al., 2005b
*Bacillus* isolates from maize rhizoplane, sampled from a commercial maize field (10 isolates)	Toxigenic *F. verticillioides* isolated from maize in Argentina	N/A	Biocontrol of *B. subtilis* against *F. verticillioides in vitro* and at the maize root level: *In vitro:* MMEA cultures: Antibiosis: dual cultures of *F. verticillioides* and *Bacillus* sp. isolates; measurement of zones of inhibition; FB_1_ levels: HPLC analyses Maize kernel cultures: Effect of *Bacillus sp*. isolates on *F. verticillioides* ergosterol content, FB_1_ accumulation and CFU counts: dual cultures of *F. verticillioides* and *Bacillus* sp. isolates; *e*rgosterol analyses as an indicator of fungal growth; determination of *F. verticillioides* CFU counts; FB_1_ levels: HPLC analyses; Greenhouse studies: *Bacillus* sp. CE1 inoculum sizes evaluated: 10^6^, 10^7^ and 10^8^ cells/ml; Effect of *Bacillus* sp. CE1 treatments on *F. verticillioides* root colonization: inoculation of seeds with different *Bacillus* sp. CE1 inoculum sizes; modified tube assay; determination of *F. verticillioides* CFU counts in the rhizoplane and endorhizosphere areas	*In vitro:* MMEA cultures: Antibiosis: significant (*P* < 0.001) inhibition (28-78%) of *F. verticillioides* growth by all *Bacillus* sp. isolates; reduction of FB_1_ levels: *Bacillus* sp. CE (50% reduction) and *Bacillus* sp. 86 (29% reduction) (*P* < 0.001); Maize kernel cultures: Ergosterol content, FB_1_ accumulation and *F. verticillioides* CFU counts were reduced by 42, 53 and 24%, respectively, after 35 days of incubation (*P* < 0.001); Greenhouse studies: *Bacillus* sp. CE1 inhibited *F. verticillioides* at the rhizoplane level at all three inoculum sizes; All bacterial treatments reduced the *F. verticillioides* CFU counts at the endorhizosphere level, the 10^8^ cells/ml treatment exhibited the highest inhibition; Good correlation (*r* = 0.995–0.998) between the antagonistic abilities of *Bacillus* sp. CE1 *in vitro* and *in planta*	Potential biocontrol agent against *F. verticillioides* infection at root level; ability to reduce *F. verticillioides* colonization of maize rhizoplane and endorhizosphere areas	Cavaglieri et al., 2005c

*N/A, Not applicable; FB_1_, Fumonisin B_1_; NOI, Niche overlapping indice; MMEA, Maize meal extract agar; PDA, Potato dextrose agar; CFU, Colony forming units*.

### Antioxidants, phenolic compounds, and essential oils

Several natural phenolic compounds derived from plants are strong antioxidants and exhibit antimicrobial activity by inhibiting the activity of key fungal enzymes, and are applied as preservatives in the cosmetic, food and drug industries (Table 3). These compounds are also considered promising antifungal agents for controlling fungal growth and associated mycotoxin production in agricultural crops pre-harvest, post-harvest, and during storage.

**Table 3:** **Current information on reduction of fumonisin-producing ***Fusarium*** spp. and fumonisin production ***in vitro*** by antioxidants/phenolic compounds and essential oils extracted from plants**.

**Biocontrol compound**	***Fusarium* spp. studied**	**Water activity (a_*w*_)**	**Test system with details of experimental model**	**Reduction criteria**	**Application**	**Reference(s)**
**ANTIOXIDANTS/PHENOLIC COMPOUNDS**						
BHA, BHT, THBP, and PP	*Fusarium verticillioides* strains RC2000, M7075, ITEM2424; *Fusarium proliferatum* strains ITEM 2443, ITEM 2444, M7089, RC2056	0.93; 0.95; 0.98; 0.995	*In vitro* control of growth and fumonisin production by *F. verticillioides* and *F. proliferatum* using antioxidants under different water availability and temperature regimes. *In vitro:* Efficacy of antioxidants: 2% MMEA prepared at different water activities; Antioxidants incorporated at 1, 5, 10 and 20 mmol.L^−1^; cultures inoculated and incubated at 18 and 25°C; measurement of the lag phase prior to growth; measurement of mycelial extension; FB_1_, FB_2_ and FB_3_ levels in MMEA cultures: HPLC analyses	*In vitro:* Efficacy of antioxidants: Control without antioxidants: increase in the lag phase of fungal growth with decreasing a_w_ and temperature; in the presence of antioxidants: increase in the lag phases of growth; no growth detected at antioxidants concentrations of 10-20 mmol.L^−1^; *F. verticillioides* and *F. proliferatum* more tolerant of THBP and BHT than PP and BHA; BHA (20 mmol.L^−1^) and PP (10 mmol.L^−1^) completely inhibited growth of both fungal species at all a_w_ levels evaluated; FB_1_, FB_2_ and FB_3_ levels in MMEA cultures: BHA (10-20 mmol.L^−1^) effective at most a_w_ levels evaluated; PP (>1 mmol.L^−1^) completely inhibited fumonisin production by both fungal species at all a_w_ levels evaluated; THBP and BHT were less effective	Food-grade preservatives BHA and PP exhibited potential for preventing mycotoxigenic fungi and their toxins entering the food chain	Etcheverry et al., 2002
BHA, BHT, THBP, and PP	*F. verticillioides* RC2000, *F. proliferatum* ITEM2443	0.95; 0.98; 0.955	Efficacy of antioxidant mixtures on growth, fumonisin production and hydrolytic enzyme production by *F. verticillioides* and *F. proliferatum in vitro* on maize-based media. *In vitro*: 2% MMEA prepared at different water activities; antioxidants incorporated at 0.5 and 1 mM; antioxidants incorporated alone and in combinations; cultures inoculated and incubated; Fungal growth: measurement of fungal extension rates; FB_1_, FB_2_ and FB_3_ levels in MMEA cultures: HPLC analyses; Hydrolytic enzyme activity: determination of N-acetyl-β-D-glucosaminidase, 2,β-D-glucosidase, and α-D-galactosidase enzyme activities with p-nitrophenyl as substrate	*In vitro*: Effect of antioxidant mixtures on lag phases and fungal growth rate: Significant (*P* < 0.001) increase in the lag phase growth of both fungal strains with BHA+PP treatment at all a_w_ levels evaluated; PP alone and in combination with BHA (0.5 and 1 mM) reduced growth rates (>85%) of both fungal species at all a_w_ levels evaluated; PP+BHT and PP+THBP treatments were less effective; FB_1_, FB_2_ and FB_3_ levels in MMEA cultures: fumonisin levels produced by both fungal species significantly (*P* <.05) reduced with BHA+PP treatments at a_w_ 0.98 and 0.955; At 0.5 mM some antioxidant treatments resulted in stimulation of fumonisin production; Hydrolytic enzyme activity: All antioxidants treatments alone and in combination resulted in significant (*P* < 0.001) reduction in total enzyme activity at all a_w_ levels evaluated	BHA and PP are permitted by the US FDA for use as antimicrobial agents in foods; BHA and PP are considered GRAS; Efficacy of BHA+PP mixtures for biocontrol of *Fusarium* spp. should be evaluated *in planta*	Reynoso et al., 2002
Commercial phenolic compounds: Benzoic acid, caffeic acid, ferulic acid; vanillic acid; Phenols extracted from plants: Chlorophorin, iroko, and maakianin	*F. verticillioides* MRC 826	N/A	Naturally occurring phenols: a detoxification strategy for FB_1_. *In vitro*: MIC of each compound: Seeded agar well diffusion technique; Effect on FB_1_ production: Alberts' broth supplemented with the respective phenolic compounds (chlorophorin at 0.45, 0.8 and 1 μmol.ml^−1^, all the other compounds at 1 μmol.ml^−1^); determination of FB_1_ levels: HPLC analyses	*In vitro:* MIC of each compound: chlorophorin, iroko, maakianin, vanillic acid and caffeic acid inhibited *F. verticillioides* growth; maakianin lowest MIC (3 μmol.ml^−1^) and therefore the most effective compound; benzoic acid and ferulic acid had no effect on fungal growth; Effect on FB_1_ production: all the compounds, except benzoic acid, reduced FB_1_ production (88–94% reduction)	Chlorophorin, iroko, maakianin vanillic acid and caffeic acid are effective in the inhibition of *F. verticillioides* growth and reduction of FB_1_	Beekrum et al., 2003
BHA and PP	*F. verticillioides* RC2000, *F. proliferatum* ITEM2443	0.95; 0.98; 0.955	Potential use of antioxidants for control of growth and fumonisin production by *F. verticillioides* and *F. proliferatum* on whole maize grain. *In vitro:* Fungal growth: rehydrated maize kernels (a_w_ 0.95, 0.98 and 0.955); antioxidants incorporated to 100, 200 and 500 μg.g^−1^ of maize; maize kernels dispensed as a monolayer in Petri dishes (a_w_ 0.95, 0.98 and 0.955); inoculation with mycelial disc; fungal colonization of grains: colony diameters; FB_1_, FB_2_ and FB_3_ levels in maize kernel cultures: HPLC analyses	*In vitro:* Fungal growth: combinations of 500 μg.g^−1^ of either BHA or PP at a_w_ 0.95 resulted in extended lag phases of fungal growth for both species; effective inhibition of growth of both fungal species by BHA and PP at 500 μg.g^−1^ at a_w_ 0.95; PP more effective than BHA; FB_1_, FB_2_ and FB_3_ levels in maize kernel cultures: fumonisin production reduced (94–98%) by BHA and PP (500 μg.g^−1^) at a_w_ 0.98; Antioxidant treatments less effective at a_w_ 0.995	BHA and PP are considered GRAS; BHA and PP effective in controlling *F. verticillioides* and *F. proliferatrum* growth and fumonisin production on maize kernels; higher concentrations needed for an effect on whole maize kernels than on MMEA Etcheverry et al., 2002, possibly due to the pericarp not allowing good contact between the fungus and the antioxidants; should be evaluated in the field as sprays	Torres et al., 2003
6,7-Dimethoxycoumarin, isolated from *Citrus sinensis* cultivar Valencia (Valencia orange)	*F. verticillioides*	N/A	Biocontrol of aflatoxins B_1_, B_2_, G_1_, G_2_, and FB_1_ with 6,7-dimethoxycoumarin, a phytoalexin from *Citrus sinensis*. *In vitro*: Induction of 6,7-dimethoxycoumarin in *Citrus sinensis* cultivar Valencia: UV irradiation of fruit; infection of fruit with *Penicillium digitatum*; Antifungal activity; FB_1_ levels: HPLC analyses	*In vitro*: Induction of 6,7-dimethoxycoumarin in *Citrus sinensis* cultivar Valencia: concentrations of 6,7-dimethoxycoumarin increased from 0.36 to 15.2 μg/g following UV irradiation; concentrations of 6,7-dimethoxycoumarin increased from 0.36 to 35.51 μg/g following infection of fruit with *P. digitatum*; Antifungal activity: 6,7-dimethoxycoumarin exhibited antifungal activity against *F. verticillioides;* FB_1_ levels: 6,7-dimethoxycoumarin caused reduction of FB_1_ production by *F. verticillioides*	-	Mohanlall and Odhav, 2006
Commercial vanillic acid and caffeic acid	*F. verticillioides* Sheldon 25N; *F. proliferatum* Matsushima Nirenberg 73N	0.88-0.97	Can phenolic compounds be used for the protection of corn from fungal invasion and mycotoxin contamination during storage? *In vitro:* Effect on fungal growth: maize kernels dispensed as a monolayer in Petri dishes; three a_w_ values (0.88–0.97) and six phenolic compound concentrations incorporated (0-2500 μg.g^−1^ maize); inoculation (mycelial disc) and incubation; measurement of colony diameters; Effect on FB_1_ production: maize kernels dispensed as a monolayer in Petri dishes; a_w_ 0.96 and a range of phenolic compound concentrations incorporated (0, 1000 and 2000 μg.g^−1^ maize); inoculation and incubation; FB_1_ levels: HPLC analyses	*In vitro*: Effect on fungal growth: increase in phenolic compound concentration positively correlated with the lag phase of fungal growth, and negatively correlated with fungal growth rate; At the highest a_w_ level evaluated (0.97) both the phenolic compounds failed to completely inhibit the growth of *F. verticillioides* and *F. proliferatum*; complete inhibition of *F. verticillioides* growth observed at a_w_ 0.921 together with vanillic acid or caffeic acid (2500 μg.g^−1^ maize); *F. proliferatum* growth completely inhibited at a_w_ 0.948 by vanillic acid (2000 μg.g^−1^ maize); caffeic acid (2000 μg.g^−1^ maize) completely inhibited *F. proliferatum* growth at a_w_ 0.921; Effect on FB_1_ production: vanillic acid (1000 and 2000 μg.g^−1^ maize) completely inhibited FB_1_ production by *F. verticillioides*; vanillic acid (2000 μg.g^−1^ maize) inhibited FB_1_ production (98% reduction) by *F. proliferatum*; caffeic acid less inhibitory than vanillic acid for both fungal species evaluated	Potential application as antifungal compounds to protect stored grains; however, high concentrations of phenolic compounds are required for efficacy on maize kernels; interaction of the phenolic compounds with maize matrix components may reduce its efficacy; high concentrations negatively affected the sensory quality of the maize; commercial application possibly not economically feasible	Samapundo et al., 2007
Commercial preparations of natural plant constituents: trans-2-hexenal; carvacrol; eugenol	*F. verticillioides* strain isolated from maize	N/A	Activity of natural compounds on *F. verticillioides* and fumonisin production in stored maize kernels. *In vitro*: Effect on conidia germination: acidified PDA; inoculation; compounds (6.2–147.6 μl/L) added to filter paper and placed inside the dish cover; incubation; determination of percentages of conidia germination; determination of MIC; Effect on mycelial growth: acidified PDA; inoculation; compounds (3.1–49.2 μl/L) added to filter paper and placed inside the dish cover; incubation; determination of percentage mycelial growth compared to the control; determination of MIC; Trials with artificially inoculated kernels: Antifungal activity in artificially inoculated maize kernels: whole maize kernels in Petri dishes; inoculation; compounds added to filter paper and placed inside the dish cover; trans-2-hexenal (24.6 μl/L), carvacrol (43.1 μl/L) and eugenol (147.6 μl/L); incubation; determination of the incidence of asymptomatic kernel infection: maize kernels from each treatment transferred to PDA; incubation; morphological identification; Effect of trans-2-hexenal treatment at different concentrations on FB_1_ and FB_2_ production in artificially inoculated maize kernels: whole maize kernels in Petri dishes; inoculation; trans-2-hexenal (92.3–369 μl/L) added to filter paper and placed inside the dish cover; incubation; determination of FB_1_ and FB_2_ levels: LC-MS/MS analyses; Trials with naturally infected kernels: Effect of trans-2-hexenal treatments on conidia germination of *F. verticillioides* in asymptomatic naturally infected maize kernels: asymptomatic naturally infected maize kernels in Petri dishes; trans-2-hexenal (92.3, 123, 184.5, 369 μl/L) added to filter paper and placed inside the dish cover; incubation; determination of the percentage infected kernels; determination of the percentage kernel germination (sprouted kernels); Semi-commercial trials: Effect of trans-2-hexenal fumigation treatment on FB_1_ and FB_2_ production by *F. verticillioides* in asymptomatic naturally infected maize kernels: trans-2-hexenal fumigation treatment (246 μl/L); maize kernels transferred to PDA; determination of FB_1_ and FB_2_ levels: LC-MS/MS analyses	*In vitro:* Effect on conidial germination: All three constituents reduced conidial germination, with trans-2-hexenal the most effective (MIC 24.6 μl/L); Effect on mycelial growth: all three constituents reduced mycelial growth, with carvacrol the most effective (MIC 24.6 μl/L); Trials with artificially inoculated kernels: Antifungal activity of the natural plant constituents in artificially inoculated maize kernels: treatments with trans-2-hexenal (24.6 μl/L), carvacrol (43.1 μl/L) and eugenol (147.6 μl/L) exhibited fungicidal activity against F. *verticillioides*; carvacrol and eugenol induced off-odors in maize; trans-2-hexenal (92.3–369 μl/L) effective in controlling the *F. verticillioides* growth (37-97% reduction); the efficacy varied with concentration and time of incubation; trans-2-hexenal (369 μl/L) induced an off-odor in maize; Effect of trans-2-hexenal treatment at different concentrations on FB_1_ and FB_2_ production in artificially inoculated maize kernels: not effective in reducing FB_1_ and FB_2_ levels; trans-2-hexenal (369 μl/L) stimulated fumonisin levels; Trials with naturally infected kernels: Effect of trans-2-hexenal treatments on conidia germination of *F. verticillioides* in asymptomatic naturally infected maize kernels: trans-2-hexenal (123-369 μl/L) reduced percentage of kernels infected with *F. verticillioides*; trans-2-hexenal (246 μl/L) provided the best control of *F. verticillioides* with no phytotoxic symptoms or off-odor; trans-2-hexenal (369 μl/L) reduced (23.3–63.3% reduction) kernel germination; trans-2-hexenal (123-246 μl/L) only delayed kernel germination; Semi-commercial trials: Trans-2-hexenal fumigation treatments: confirmed efficacy of reducing fungal infection; trans-2-hexenal fumigation treatments failed to reduce fumonisin levels	Trans-2-hexenal effective in controlling *F. verticillioides* growth, also in asymptomatic maize kernels; trans-2-hexenal as fumigant penetrates into the internal part of maize kernels	Menniti et al., 2010
Aqueous and organic extracts of weedy plants collected in the Gauteng and North West Provinces of South Africa: *Tagetes minuta;* *Lippia javanica;* *Amaranthus spinosus*; and *Vigna unguiculata*	*F. verticillioides* (MRC 826, 8267, 8559); *F. proliferatum* (MRC 2301, 6908, 7140)	N/A	Antifungal activity of four weedy plant extracts against selected mycotoxigenic fungi. *In vitro*: Preparation of plant extracts: Drying of aerial parts of plants at room temperature; grinding of dried plants into a powder; sequential extraction with hexane, dichloromethane, methanol and water; drying of extracts; Determination of the MIC: Serial dilution microplate technique	*In vitro*: Inhibition of fungal growth: water extracts of all four plant species exhibited no antifungal activity at the highest concentration (2.5 mg.ml^−1^); methanol, hexane and dichloromethane extracts of *A. spinosus* and *V. unguiculata* exhibited the broadest spectrum antifungal activity after 48 h; all solvent extracts of *A. spinosus* and *V. unguiculata* exhibited the highest inhibitory and stability effects over 120 h against all *Fusarium* strains. Stability of plant extracts over 120 h: dichloromethane extracts loses its activity more rapidly than methanol and hexane extracts	Extracts of *V. unguiculata* and *A. spinosus* could potentially be applied in crop disease management; development of cost-effective biofungicides for application in rural subsistence farming communities	Thembo et al., 2010
THC compounds: THC1, THC2 and THC3 [Natural THC compounds are extracted from the roots of *Curcome longa* L. (Turmeric)]	*F. proliferatum* INRA 212	N/A	*In vitro* inhibitory effect of tetrahydrocurcuminoids on *F. proliferatum* growth and FB1 biosynthesis. *In vitro:* Antifungal activity of THC1: THC1 (2.7, 8.1, and 13.4 μmol.ml^−1^ THC1) solution distributed on surface of PDA plates and air dried; inoculation with *F. proliferatum* and incubation; determination of fungal growth: measurement of colony diameter; determination of inhibition percentage: radial growth in relation to the control; comparison with results from THC2 and THC3; Effect on FB_1_ levels: Cultivation in GYEP liquid medium; inoculation; culture supplemented with THC1 (0.8, 1.3, 1.9, 2.7 μmol.ml^−1^); incubation; FB_1_ levels: HPLC analyses	*In vitro:* Antifungal activity of THC1: Fungal growth decreased significantly (*P* < 0.05) with increasing concentration of THC1 (2.7 to 13.4 μmol.ml^−1^); THC1 (13.4 μmol.ml^−1^) exhibited the highest percentage inhibition (70%); Effect on FB_1_ levels: FB_1_ production reduced in the presence of THC1, THC2, and THC3; FB_1_ levels 35, 50 and 75% reduced by THC1 at 0.8, 1.3, and 1.9 μmol.ml^−1^, respectively; THC1 (2.7 μmol.ml^−1^) treatment resulted in complete inhibition of FB_1_ production	THC compounds are promising biocontrol agents due to low inhibitory concentrations; THC1 is a food-grade compound and can be produced on large scale for industrial application	Coma et al., 2011
Extracts of *Gynostemma pentaphyllum* (Southern Ginseng)	*F. verticillioides*	N/A	Antimicrobial activity of *G. pentaphyllum* extracts against fungi producing aflatoxin and fumonisin and bacteria causing diarrheal disease. *In vitro:* Antifungal activity	*In vitro*: Antifungal activity: extracts exhibited antifungal activity against *F. verticillioides* growth (41-43% reduction)	*G. pentaphyllum* is frequently being applied as herbal medicine; extracts could be applied to control *F. verticillioides* growth	Srichana et al., 2011
70% Ethanol extracts of *Equisetum arvense* (Horsetail) and *Stevia rebaudiana* (Candyleaf)	*F. verticillioides* (UdL-TA 3.215)	0.93-0.95	Effect of extracts on growth and mycotoxin production by *A. flavus* and *F. verticillioides* in maize seeds as affected by water activity. *In vitro*: Fungal growth: preparation of maize kernels (a_w_ levels adjusted to 0.93 and 0.95, respectively) and supplementation with plant extracts, separately and in 1:1 mixtures, respectively; maize kernels in single layers in Petri dishes; inoculation and incubation; determination of CFU counts after 10, 20 and 30 days of incubation by employing a selective medium for *Fusarium* spp.; FB_1_ and FB_2_ levels in maize cultures: HPLC analyses	*In vitro*: Effect of plant extracts on fungal growth: extracts of *S. rebaudiana* significantly reduced CFU counts of *F. verticillioides;* (>99% reduction; a_w_ 0.95); *E. arvense* reduced CFU counts of *F. verticillioides* at a_w_ levels 0.93 and 0.95), but not as effective as *S. rebaudiana*; significant (*P* < 0.05) stimulation of growth observed in a few cases; FB_1_ and FB_2_ levels in maize cultures: fumonisin production was not significantly affected	-	Garcia et al., 2012
**ESSENTIAL OILS**						
Essential oils extracted from cinnamon, clove, oregano, palmarose and lemongrass	*F. proliferatum* (three different isolates)	0.95 and 0.995	Inhibitory effect of cinnamon, clove, lemongrass, oregano and palmarose essential oils on growth and FB_1_ production by *F. proliferatum* in maize grain. *In vitro:* Effect of essentials oils on growth rate and FB_1_ production by *F. proliferatum*: Preparation of maize kernel medium: gamma irradiation of dent maize kernels; rehydration of maize kernels to a_w_ 0.95 and 0.995; addition of essential oils to maize kernels (final concentration 500 and 1000 μg.g^−1^of maize); equilibration; Culture conditions: single layer of maize kernels in Petri dishes; inoculation (agar disk method); variables: essential oil concentration; water activity; temperature (20 and 30°C), fungal isolates; Fungal growth: measurement of colony diameter; FB_1_ levels in maize cultures: HPLC analyses	*In vitro:* Effect of essentials oils on growth rate of *F. proliferatum*: All five essential oils had a significant (*P* < 0.05) inhibitory effect on growth of *F. proliferatum* at a_w_ 0.995 at both temperatures; At a_w_ 0.95, the effect of essential oils on growth rates was dependent on the temperature; incubation at 20°C: oil of cinnamon, clove and oregano (1000 μg essential oil.g^−1^ of maize) had a significant (*P* < 0.05) inhibitory effect on *F. proliferatum* growth; at concentrations of 500 μg essential oil.g^−1^ of maize only cinnamon and oregano were effective; incubation at 30°C: none of the essential oils analyzed had an inhibitory effect on any of the fungal growth rates; FB_1_ levels in maize cultures: At a_w_ 0.995 and both temperatures, cinnamon, oregano and palmarose oils had a significant (*P* < 0.05) inhibitory effect on FB_1_ production by all three fungal strains; clove and lemongrass oils only exhibited a significant inhibitory effect at 30°C; At a_w_0.950 none of the essential oils had a significant effect on FB_1_production; essential oil concentration did not affect FB_1_ production	Cinnamon and oregano oils could be effective in controlling growth and FB1 production by *F. proliferatum* in maize pre-harvest	Velluti et al., 2003
Essential oils and oleoresins extracted from *Zingiber officinale* (Ginger); Synthetic antioxidants BHA, BHT and PG	*F. verticillioides* (*= F. moniliforme*)	N/A	Chemistry, antioxidant and antimicrobial investigations on essential oil and oleoresins of *Z. officinale*. *In vitro*: Extraction from *Z. officinale* rhizomes: essential oils were extracted by hydro distillation; oleoresins were extracted with ethanol, methanol, carbon tetrachloride and isooctane, respectively; Phytochemistry and identification of extracted components: GC-MS; Antioxidant activity of components compared with BHA, BHT and PG: peroxide-, anisidine- and thiobarbituric acid values; DPPH radical scavenging and total antioxidant activity by ferric thiocyanate methods; Antifungal activity: “Poisoned food” technique: ginger oil and oleoresins (2, 4, and 6 μl) mixed with CDA culture medium and poured into Petri plates; inoculation (mycelial discs) and incubation; measurement of radial growth: average colony diameters; calculation of the percentage mycelial zone inhibition; Inverted Petri plate technique: CDA Petri dishes inoculated with fungi; Petri dishes inverted; filter paper disks soaked with ginger oil and oleoresins, respectively and placed inside inverted lids; incubation; calculation of the percentage mycelial zone inhibition	*In vitro*: Extraction from *Z. officinale* rhizomes: a large number of components extracted; major components: geranial (essential oil), eugenol (ethanol oleoresin extract) and singerone (methanol, carbon tetrachloride and isooctane oleoresin extracts); Antioxidant activity of components compared with BHA, BHT and PG: the presence of the essential oils, oleoresins and antioxidants resulted in reduced peroxide- and anisidine values and DPPH radical concentration; antioxidant activity of essential oils and oleoresins is comparable to BHA and BHT, but less than PG; the essential oils and ethanol oleoresin extracts exhibited better antioxidant activity than other oleoresins and the synthetic antioxidants; Antifungal activity of components: essential oils and oleoresins moderate to good inhibition *F. verticillioides* growth; ginger oil and the CCl_4_ oleoresin extract (6 μl dose of each) highly effective against *F. verticillioides* growth (100% inhibition); Essential oils generally more effective than the oleoresins	Preservation of edible oils and other foodstuffs against autoxidation and microbial spoilage	Singh et al., 2008

*BHA, Butylated hydroxyanisole; BHT, Butylated hydroxytoluene; THBP, Trihydroxybutyrophenone; PP, Propylparaben; PG, propyl gallate; DPPH, 1,1-Diphenyl-2-picrylhydrazyl; FB_1_, Fumonisin B_1_; FB_2_, Fumonisin B_2_, FB_3_, Fumonisin FB_3_; US FDA, United States Food and Drug Administration; GRAS, Generally regarded as safe; MIC, Minimum inhibitory concentration; UV, Ultra violet; THCs, Tetrahydrocurcuminoid compounds; MMEA, Maize meal extract agar; CDA, Czapek-Dox agar*.

### Antioxidants

The food-grade antioxidants butylated hydroxyanisole (BHA) and propylparaben (PP) have shown potential for controlling *F. verticillioides* and *F. proliferatum* growth and fumonisin production at a variety of water activities and incubation temperatures *in vitro* (Etcheverry et al., 2002; Table 3). Both fungal species were more sensitive to BHA and PP than the other antioxidants evaluated, i.e., trihydroxybutyrophenone (THBP) and butylated hydroxytoluene (BHT). In another study, combination treatments of BHA and PP resulted in further reduction of fumonisin production (Reynoso et al., 2002). BHA, PP, and BHT alone or in combination also resulted in a significant (*P* < 0.001) reduction in hydrolytic enzyme activity, which is required for early fungal growth. Similar results were reported by Torres et al. (2003). BHA is produced naturally by *Botryococcus braunii, Cylindrospermopsis raciborskii, Microcystis aeruginosa*, and *Oscillatoria* sp., while PP is a natural compound extracted from plants. Both antioxidants are also produced synthetically, are considered GRAS by the US FDA and frequently employed as preservatives in the food and cosmetic industries (Reynoso et al., 2002; Rawal et al., 2010; US FDA, GRAS substances evaluated by SCOGS).

Tetrahydrocurcuminoids (THC), a class of phenolic antioxidants extracted from the roots of the non-toxic herbaceous plant *Curcoma longa* L. (Turmeric), inhibits *F. proliferatum* growth and FB_1_ production *in vitro* (Coma et al., 2011; Table 3). THC1, a food-grade compound containing two guaiacyl phenolic subunits, exhibited high antifungal activity and inhibition of FB_1_ production in liquid cultures at low inhibitory concentrations. FB_1_ production was affected irrespective of the effect on fungal growth, indicating that fungal growth and FB_1_ biosynthesis are independently modified by THC1. Comparative studies on THCs and related molecules *n*-propylguaiacol, eugenol, acetylacetone, and ferulic acid indicated that the presence of the benzene rings and guaiacyl groups play an important role in fungal inhibition (Beekrum et al., 2003; Samapundo et al., 2007). It was further noticed that the presence of hydroxyl and methoxy groups in the *ortho* position of the benzene ring of THC molecules affects the degree of antifungal activity, while the enolic part of the non-phenolic THC3 molecule could play a role in bioactivity. It was suggested that the biochemical mechanisms involved during antioxidant and antifungal activities differ between the respective THC compounds, as the presence of a phenol group in the meta- or para-position of the linking chain and a phenol or a methoxy group adjacent to it is required for antioxidant activity.

### Phenolic compounds

Investigations into the effects of the natural phenolic compounds vanillic and caffeic acid on *F. verticillioides* and *F. proliferatum* growth and FB_1_ production at different water activities in maize *in vitro* indicated that an increase in phenolic compound concentration results in an increase in the lag phase of growth, and a decrease in fungal growth rate and FB_1_ production (Samapundo et al., 2007; Table 3). In general, complete inhibition of *Fusarium* growth was observed at relatively high phenolic concentrations and low water activities. *F. proliferatum* was more sensitive, exhibiting complete inhibition of growth in the presence of the compounds. Both compounds significantly reduced FB_1_ production by *F. verticillioides* and *F. proliferatum*, with vanillic acid being more effective. No FB_1_ was produced by *F. verticillioides* in the presence of vanillic acid at the lowest concentration tested.

*F. verticillioides* growth and FB_1_ production are inhibited by several other plant phenolic compounds *in vitro* (Table 3). Chlorophorin, iroko, maakianin, vanillic acid, and caffeic acid inhibits *F. verticillioides* growth, while FB_1_ production is inhibited by chlorophorin, iroko, vanillic acid, caffeic acid, and ferulic acid (Beekrum et al., 2003; Table 3). Flavonoids, phenolic acid, and terpine rich 70% ethanol extracts of the non-toxic food-grade plants *Equisetum arvense* (Horsetail) and *Stevia rebaudiana* (Candyleaf), effectively inhibited *F. verticillioides* growth, with *S. rebaudiana* being more effective (Garcia et al., 2012). However, fumonisin production was not affected. Extracts of the herbaceous climbing vine of the family Cucurbitaceae, *Gynostemma pentaphyllum* (Southern Ginseng), inhibited growth of *F. verticillioides* (Srichana et al., 2011). *G. pentaphyllum* is frequently applied as herbal medicine and exhibits high antioxidant activity. Fumigation by trans-2-hexanal (extracted from fruits and vegetables), carvacrol (extracted from oregano and thyme), and eugenol (extracted from cinnamon and clove) effectively inhibits *F. verticillioides* conidial germination and mycelial growth in maize kernels, with trans-2-hexanal the most effective (Menniti et al., 2010). Trans-2-hexanal fumigation was also effective in controlling the fungus in asymptomatic kernels. However, the treatment does not reduce fumonisin levels post-harvest, but reduces the germ-ability of maize kernels. The compound 6,7-dimethoxycoumarin, occurring in *Penicillium digitatum* infected *Citrus sinensis* cultivar *Valencia* fruit (Valencia orange), reduces *F. verticillioides* growth and FB_1_ production (Mohanlall and Odhav, 2006). Possible mechanisms of inhibition by phenolic plant extracts include disruption of the fumonisin biosynthetic pathway; effects on colony morphology; granulation of the cytoplasm; and rupture of the cytoplasmic membrane (Garcia et al., 2012).

### Essential oils

Essential oil and oleoresins extracted from *Zingiber officinale* (Ginger) rhizomes exhibit clear antimicrobial activity against *F. verticillioides* (= *F. moniliforme*) *in vitro* (Singh et al., 2008; Table 3). Ginger oil and carbon tetrachloride oleoresin extracts have shown highly effective inhibition of *F. verticillioides* growth. The antioxidative potential of the essential oil and oleoresins, in terms of peroxide content, anisidine and thiobarbituric acid values, 1,1-diphenyl-2-picrylhydrazyl free radical scavenging activity and total antioxidant activity was in general comparable to the antioxidants BHA and BHT, but not as effective as propyl gallate. The phenolic compound geranial is dominant in the essential oil component, while eugenol and singerone are dominant in the oleoresin extracts. The antioxidant activity could also be enhanced by a possible synergistic effect of the phenolic compounds.

Essential oils extracted from cinnamon, clove, oregano, palmarosa and lemongrass inhibit growth and FB_1_ production by *F. verticillioides* and *F. proliferatum in vitro* (Velluti et al., 2003; Table 3). The inhibitory effect of the essential oils was overall more pronounced at higher water activities, probably due to more effective penetration of oils into kernels in the presence of water. The antimicrobial activity of these oils could be attributed to the presence of aliphatic alcohols and phenols in their chemical composition. Oils of cinnamon and oregano were most promising for control of fungal growth and FB_1_ production by *F. proliferatum*, and cinnamon, oregano and lemongrass oils for *F. verticillioides*. These oils could be effective in controlling fungal growth and FB_1_ production in maize under pre-harvest conditions.

### Developing resistant crops through breeding and genetic engineering

Studies in breeding and genetic engineering for resistance in crops are mainly aimed at preventing invasion by insects, contamination by mycotoxigenic fungi and detoxification of mycotoxins *in planta* through various molecular strategies (Duvick, 2001; Cleveland et al., 2003). Selection of resistant genotypes is complex, it requires sufficient genotypic variation within the breeding material; is affected by climatic conditions; and should be tested across several locations and years (Löffler et al., 2010). Lower mycotoxin levels measured in United States and Canadian maize, where no fungicide was introduced, was attributed to successes with breeding resistant maize varieties.

Extensive genomic resources are essential for investigations into the biochemical and regulatory pathways of mycotoxin biosynthesis, pathogenesis of fungal–plant interactions, and the development of targeted and innovative approaches for breeding and engineering crops for resistance (Cleveland et al., 2003; Brown et al., 2006; Desjardins and Proctor, 2007). Whole genome sequences and expression sequence tags (ESTs) are important tools for understanding disease caused by fungi, fungal lifecycles and secondary metabolism. Available genomic resources include genetic maps, genome sequences, an EST library, and an integrated gene index. Next-generation RNA sequencing was used to study transcriptional changes associated with *F. verticillioides* inoculation in resistant and susceptible maize genotypes by including an extensive range of maize inbred lines (Lanubile et al., 2014). The technique generated extremely useful data on genetic markers involved in recognition, signaling, and controlling host resistance mechanisms. It also provided quantification of expression, thus enabling interpretation of defense responses. The data provides an important genomic resource for the development of disease resistant maize genotypes. Genetic markers identified through this technique could be added to existing information on single nucleotide polymorphism markers.

### Natural resistance in crops

Comprehensive knowledge on the biochemical and molecular mechanisms involved in natural resistance of crops is imperative for the further development of resistance to *Fusarium* infection and insect infestation in crops (Cleveland et al., 2003). The whole genome sequence of maize is available (Schnable et al., 2009), permitting genome-wide expression analysis of the maize–*Fusarium* interaction. Studying maize varieties with varying degrees of resistance enables researchers to associate resistant crops with specific genetic, biochemical and anatomical traits. Regions on chromosomes associated with natural resistance to insect invasion, fungal contamination, or mycotoxin production are identified, resistant traits mapped and resistant lines crossed with commercially acceptable lines. Chromosomal regions could be associated with resistance to fungal growth; with mycotoxin production; or with both traits, indicating the possibility of separate genetic control (Cleveland et al., 2003). Comparison of kernel protein profiles between susceptible and resistant genotypes through proteomic analyses contributes to identifying resistance associated proteins. Resistant inbred lines are distinguished from susceptible lines and serve as sources of resistant germplasm.

Expression profiles for maize genes during infection with *F. verticillioides* indicated up-regulation of genes encoding a range of proteins related to cell rescue, defense, and virulence in both resistant and susceptible maize lines, including pathogenesis related (PR) proteins [e.g., chitinase (reducing chitin in fungal membrane); permatin (fungal hyphae leak and rupture)]; proteins involved in detoxification response (e.g., cytochrome P450 monoxygenase, peroxidases, and glutathione-S-transferases); heat-shock proteins (regulating folding of resistance proteins); and proteinase inhibitors (Lanubile et al., 2010). Resistance in maize lines could be due to constitutive defense mechanisms that resist fungal infection (Lanubile et al., 2010; Campos-Bermudez et al., 2013). In resistant maize lines defense-related genes, encoding constitutively expressed PR, detoxification enzymes, and β-glucosidases, were transcribed at high levels before infection, and provided defense against the fungus. In susceptible maize lines, defense genes are induced as a response to pathogen infection, though not sufficiently enough to prevent progress of the disease.

Host–pathogen recognition and interaction processes underlie resistance and susceptibility (Campos-Bermudez et al., 2013). Sucrose is one of the compounds that play an important role in host-pathogen recognition and in the outcome of interactions. During fungal infection plant carbohydrate metabolism is manipulated by induced invertase and sucrose synthase enzymes and the formation of hexoses required for fungal growth. Maize lipoxygenase (*ZmLOX*) derived oxylipins (e.g., jasmonic acid) are known for regulating plant defense against pathogens, and also play an important role in recognition during host-pathogen interactions, as indicated by up-regulation of LOX genes *ZmLOX5* and *ZmLOX12* in a response to *F. verticillioides* infection (Maschietto et al., 2015).

Mapping of chromosomal regions encoding *Fusarium* ear mold resistance as quantitative trait loci (QTL) and the employment of marker-assisted QTL in selection for *Fusarium* ear mold resistance are valuable tools being developed for maize hybrid development (Duvick, 2001). Ear mold resistance can be mapped as QTL using large segregating plant populations. Molecular markers linked to these QTL could be valuable during inbred development. Other factors that enhance the susceptibility of maize genotypes include: late-maturing cultivars where grain moisture content decreases slowly; upright cobs and thin grain pericarp which increase susceptibility to fungal infection; tightness of husks; and the competitive advantage of *F. verticillioides* by having a broader optimum temperature range than *F. graminearum* (Butròn et al., 2006).

### Genetic engineering for resistance to insect infestation and *Fusarium* infection in crops

Natural fungal and insect resistance mechanisms could be further enhanced in commercially acceptable crops through genetic engineering (Cleveland et al., 2003). The role of hemicellulose, cysteine protease, peroxidase, α-amylase inhibitors, as well as maize ribosomal inactivating protein in insect resistance mechanisms are important focus areas. Genetically modified *Bt* maize expressing *cry* proteins from the bacterium *Bacillus thuringiensis*, has the potential to reduce insect damage and fumonisin levels compared to non-*Bt* hybrids. Furthermore, chitinase enzymes for digestion of chitin, an integral part of the exoskeleton of insects, have been applied for control of *Sesamia cretica* (corn borer; Osman et al., 2015). A chitinase gene from the cotton leaf worm, *Spodoptera littoralis*, was expressed in transgenic maize, and resulted in enhanced resistance against *S. cretica*. The development of transgene resistance to fungal disease appears to be more challenging than insect resistance (Duvick, 2001). Although, moderate resistance was demonstrated in model systems, no transgenic crops with effective resistance to fungal disease are commercially available. However, genetics of *Fusarium* infection of maize kernels, development of disease symptoms and biosynthesis of fumonisins is a rapid developing field and could provide more insights for developing transgenic resistance to *Fusarium* infection in the near future.

Genetic engineering approaches include the cloning and expression of genes encoding maize secondary metabolites with antifungal properties and the overexpression of pathway-limiting enzymes (Duvick, 2001). However, it should be kept in mind that diversion of metabolic pathways could compromise other vital biosynthetic routes. Expression of antifungal protein in tissue critical for fungal infection could be a strategy, while different types of resistance could be employed by pyramiding different types of resistance genes into commercial germplasm. Host plant–pathogen interactions are complex, involving multiple proteins and metabolites as well as competition for biomass and nutrients. Signaling pathway genes control a variety of cellular defense pathways involving protein-protein interactions. Engineering of the main signals controlling defense gene expression could result in more effective defense response including constitutive response or a chemically induced response and the development of enhanced disease resistance phenotypes.

Another approach involves the expression of catabolic enzymes to detoxify mycotoxins *in situ* before it accumulates in the plants (Duvick, 2001). Success depends on several factors: the extent to which the plant-produced enzyme reaches its target substrate and the stability of the detoxification step; enzyme localization in the seed in relation to mycotoxin accessibility; kinetic parameters of the enzyme in the context of its localization in the plant; stability and activity of the enzyme pre- and post-harvest; and the identity and toxicity of breakdown products.

### *Bt* maize

Genetic modification of maize plants to express insecticidal Cry proteins of *Bacillus thuringiensis* (called *Bt* maize) provides a safe and highly effective method for insect control and accompanying *Fusarium* infection and fumonisin production (Betz et al., 2000). Corn borers cause considerable damage to maize stalk and ear tissue, which in turn stimulates germination of *F. verticillioides* spores, leading to progressive ear and kernel rot and eventually production of increased levels of fumonisins. A significant correlation was reported between the extent of insect damage and total fumonisin levels in maize (Dowd, 2001). Cry1Ab protein in *Bt* protected maize reduces corn-borer damage in maize dramatically, resulting in considerable less *Fusarium* infection and reduced fumonisin levels (Betz et al., 2000). Cry proteins are selectively active against a specific range of insects including lepidopteron and coleopteran insect pests. Extensive field trials across the USA and Europe confirmed frequently lower fumonisin concentrations detected in maize using *Bt* maize hybrids (Hammond et al., 2004), thereby increasing the percentage maize grain suitable for human consumption. In South Africa, there has been a decrease over the last 20 years in the amount of chemical insecticides used, due to the cultivation of *Bt* crops (Kunert, 2011). In the US States the annual benefits that *Bt* maize provides in terms of lower fumonisin and aflatoxin contamination are estimated at about $23 million (Wu, 2006). *Bt* maize could especially be a useful tool in developing countries.

The insecticidal nature of the Cry proteins has led to the development of a variety of commercial *Bt* microbial pesticide products since 1961 (Betz et al., 2000). Extensive toxicological studies by the US Environmental Protection Agency (EPA) and the World Health Organisation (WHO) have proven the safety of *Bt* protected crops and products to humans, animals and the environment [US EPA, 1998a,b; International Programme on Chemical Safety (IPCS), 1999]. Food derived from *Bt* crops has also been fully approved by numerous regulatory agencies through-out the world. Safety considerations were further supported by the more than 50 years history of safe use of these products (McClintock et al., 1995). The potential for human and non-target exposure is extremely low, as Cry proteins exhibit a high degree of specificity toward the target insect species, should be ingested to activate in the target species and should have no contact activity (Betz et al., 2000). *Bt* products are considered to reduce the risks posed by insecticides, thereby impacting less on the environment. It also functions as a supplementary pest control by enhancing the presence of beneficial natural occurring non-target insects (Gianessi and Carpenter, 1999). The cultivation of *Bt* protected maize by growers increased rapidly throughout the world since its commercial introduction in 1996 (Betz et al., 2000). Grower approval could be ascribed to increased crop yields, reduced crop damage and input costs as a result of reduction in the use of chemical pesticides; and highly effective pest control. Cry proteins in the plant tissue are not affected by application timing, accuracy of application, concentration, rain or sunlight. *Bt* crops are entirely equivalent to non-recombinant plants, except for the presence of *cry* genes and proteins. *Bt* protected crops and products meet important standards for biological control agents regarding technical viability, need, safety and efficacy.

Recently, increasing insect resistance and accompanied occurrence of resistance alleles in insects against first generation *Bt* crops have been reported (Kunert, 2011; Abbas et al., 2013). Efforts to reduce the development of target insect resistance to *Bt* crops include introduction of a refuge strategy, which involves the cultivation of non-*Bt* crops nearby *Bt* crops to prevent domination of resistant insect species. The effectiveness of *Bt* crops is also influenced by fluctuation of the *Bt* protein concentrations produced in plants, which in turn is determined by factors such as plant maturation and photosynthesis. Possible structural changes of *Bt* proteins, including changes in micro-RNA and protein profiles were also reported. *Bt* maize genotype plays a determining role in the efficacy of insect damage control (Clements et al., 2003). *Bt* (Cry1Ab protein) protected plants could reduce fumonisin concentration in maize during seasons when the European corn borer (*O. nubilalis* Hübner) dominates, but not in seasons when the corn earworm (*H. zea* Boddie) dominates. Tende et al. (2010) evaluated sensitivity of the stalk borer species *Chile partellus* (Lepidoptera, Crambidae) and *Busseola fusca* (Lepidoptera, Noctuidae) toward endotoxins constitutively produced by two *Bt* maize inbred lines frequently cultivated in Kenya. The *Bt* maize inbred lines (Event 223 *cry1AB::Ubiquitin* and Event 10 *cry1Ba::Ubiquitin*) reduced *C. partellus* survival significantly and sensitivity remained constant through eight generations. However, *B. fusca* invasion could not be sufficiently controlled by these inbred lines and remained unchanged through five generations. More efficient transgenic *Bt* crops could be produced through gene pyramiding (Kunert, 2011).

## Post-harvest biologically based control methods for reduction of the fumonisins in food and feed

### Natural clay adsorbents

Introduction of natural clay adsorbents during food processing leads to detoxification of contaminated food through adsorption of mycotoxins (Aly et al., 2004; Robinson et al., 2012). The bioavailability of mycotoxins in animal feed is also reduced in this manner, thereby preventing toxic interactions and absorption across the gastrointestinal tract.

Montmorillonites are a group of phyllosilicate clay minerals that have the ability to adsorb organic compounds through cation-exchange (Aly et al., 2004). The adsorption abilities of montmorillonite clays are higher than other clay minerals due to their large molecular structure and surface area that increases considerably when wet. Their chemical structures are characterized by alternating layers of tetrahedral silicon and octahedral aluminum coordinated with oxygen atoms. Montmorillonite clay minerals effectively reduce FB_1_ in aqueous solutions *in vitro*, and in human- and animal models *in vivo* through adsorption (Table 4). The adsorption is saturable and occurs largely within the interlaminar regions of the clay (Mitchell et al., 2013). Certain clay minerals, particularly naturally occurring aluminum oxides have structure-selective affinities for different mycotoxins and the degree of adsorption depends on the polarity of the molecules, while the particle size of clays could also influence binding affinity (He and Zhou, 2010). A correlation exists between the binding capacity of the clays and the ratio of their surface acidity to pore volume. In this regard, the slightly higher adsorption of AFB_1_ than FB_1_ to hydrated sodium calcium aluminum magnesium silicate hydroxide (Egyptian montmorillonite, EM) and hydrated sodium calcium aluminum silicate (HSCAS) in spiked malt extracts, could be ascribed to the difference in polarity between the molecules (Aly et al., 2004). The adsorption capacity of montmorillonite clays can be enhanced by addition of phosphate and polyphosphate salts, bentonite, or calcined attapulgite (He and Zhou, 2010). A combination of clay minerals (1–10%) and modified yeast cell wall extracts (90–99%) could be beneficial for adsorption of multiple mycotoxins, including the fumonisins (Howes and Newman, 2000).

**Table 4:** **Current information on reduction of fumonisin B_**1**_ in aqueous solutions (***in vitro***), and human and animal models (***in vivo***) through adsorption to clay minerals**.

**Clay mineral**	**Test system with details of experimental model**	**Reduction criteria**	**Application**	**Reference(s)**
HSCAS; EM	Application of adsorbent agent technology in the removal of AFB_1_ and FB_1_ from malt extract. *In vitro*: Adsorption ability of HSCAS and EM for FB_1_ in aqueous solutions: Adsorbents (0.5; 1; 2; 4% w/v) weighed out in glass tubes; FB_1_ added (5, 10 and 50 ppm in aqueous solution); reaction: 1 h at 25°C; centrifugation; determination of FB_1_ levels in the supernatant; Adsorption ability of HSCAS and EM for FB_1_ in aqueous malt extract: preparation of FB_1_ contaminated malt (50, 100 and 200 ppm FB_1_); preparation of malt extract: steeping of spiked malt extract; collection of steep; addition of HSCAS and EM (0.5 % w/v); shaking for 30 min; centrifugation; filtration; determination of FB_1_ levels in filtrate; FB_1_ levels: HPLC analyses	*In vitro*: Adsorption ability of HSCAS and EM for FB_1_ in aqueous solutions: Both sorbents (0.5% w/v) exhibited high affinity to adsorb to FB_1_ in aqueous solutions at different contamination levels: Adsorption ability of HSCAS and EM for FB_1_ in aqueous solutions: adsorption ability of HSCAS 85.1-92.4%; adsorption ability of EM 78.2–92.2%; lower levels of adsorbents (0.5%) resulted in more effective adsorption; Adsorption ability of HSCAS and EM (both 0.5% w/v) for FB_1_ in aqueous malt extract: adsorption ability of HSCAS 85.25–91.97%; adsorption ability of EM 88.4–92.47%	Food and beverage industries: removal of FB_1_ from aqueous solutions, i.e. during the extraction of malt	Aly et al., 2004
NS (Novasil)	Calcium montmorillonite clay reduces urinary biomarkers of FB_1_ exposure in rats and humans. *In vivo:* Rodent model: Male Fisher 344 rats; FB_1_ and NS added to feed; treatment groups: absolute control, FB_1_ control, and FB_1_ plus NS (2% w/w); acclimation period; FB_1_ dosage (25 mg/kg bw) based on an average of 150 g bw; supplemented feed administered to rats by single aqueous gavage; urine samples collected daily; Human study: participants recruited from six communities within the Ejura-Sekyedumase district of Ghana; three study groups: High dose (NS 3 g/day; low dose (NS 1.5 g/day) and placebo control; study period: 3 months; collection of urine at multiple time points; UFB_1_ biomarker levels: HPLC analyses; Creatinine levels in urine samples: MALDI-TOF MS	*In vivo:* Effect of dietary NS on UFB_1_ levels in rats and humans: NS significantly reduced the excretion UFB_1_ in urine: Rodent model: NS treatment significantly reduced UFB_1_ by 20% in 24 h and 50% after 48 h; Human study: week 8 and 10 high and low dose NS treatments resulted in decreased percentage of participants with detectable UFB_1_; median levels of the high dose group at week 8 were significantly (*P* < 0.05) lower than the placebo group; week 10 median UFB_1_ levels for both high and low dose groups were significantly (*P* < 0.05) reduced	Reduction of fumonisin exposure in communities at risk in Ghana: NS could be a suitable enterosorbent for reduction of the bioavailability of fumonsins in the gastrointestinal tract of animals and humans; intervention methods in the form of capsules or other dose forms; further studies: to determine whether a time-related effect exists, to confirm the efficacy and safety of NS clay as a multifunctional intervention and to determine the nutritional implications of NS supplementation of diets	Robinson et al., 2012
Refined UPSN, particle size 45-100 μm	Calcium montmorillonite clay reduces AFB_1_ and FB_1_ biomarkers in rats exposed to single and co-exposures of aflatoxin and fumonisin. *In vivo*: Rodent model: Male Fisher 344 rats; treatment groups: absolute control, FB_1_ (25 mg/kg bw) treatment, AFB_1_ (25 mg/kg bw) treatment and FB_1_ (25 mg/kg bw) + AFB_1_ (0.125 mg/kg bw) treatment; FB_1_, AFB_1_ and FB_1_+AFB_1_ treatment groups were supplemented with UPSN (0%, 0.25%, and 2%); acclimation period; supplemented feed administered to rats by single aqueous gavage; collection of urine at multiple time points over 72 h; UFB_1_ levels: HPLC analyses	*In vivo*: FB_1_: FB_1_ treatment: UPSN (2% w/w) significantly (*P* < 0.0001) decreased UFB_1_ levels at 12, 24 and 36 h; 2% UPSN treatment more effective than the 0.25% UPSN treatment: 2% UPSN treatment 85 and 98% reduction at 12 and 24 h, respectively; 0.25% UPSN treatment 45 and 55% reduction at 12 and 24 h, respectively; AFB_1_/FB_1_ co-treatment: Lower efficacy than with separate UPSN treatments; a dose-dependent reduction in UFB_1_ for the UPSN treated AFB_1_/FB_1_ groups: 2% UPSN more effective than the 0.25% UPSN treatment: 2% UPSN treatment 51 and 59% reduction at 12 and 24 h, respectively; 0.25% UPSN treatment 28 and 39% reduction at 12 and 24 h, respectively; 2% UPSN treatment: significant reduction at 12 h (*P* < 0.0177), 24 h (*P* < 0.0284 and 72 h (*P* < 0.0001); 0.25% UPSN treatment: reduction only statistically significant at 72 h (*P* < 0.0369); AFB_1_: UPSN treatment reduced AFM_1_ biomarkers in a dose-dependent manner with the largest reduction in the 2% treatment group (97 and 99% reduction after 12 and 24 h, respectively); AFB_1_/FB_1_ co-treatment: Lower efficacy than with separate UPSN treatments; UPSN treatment dose-dependently reduced AFM_1_ excretion; 0.25% UPSN treatment more effective than the 2% treatment group	Economical and sustainable intervention to reduce exposure to FB_1_ and AFB_1_; utilization of the clay as a binder for both FB_1_ and AFB_1_; application could selectively reduce levels below carcinogenic thresholds	Mitchell et al., 2013

*HSCAS, Hydrated sodium calcium aluminum silicate; EM, Egyptian montmorillonite (Hydrated sodium calcium aluminum magnesium silicate hydroxide); NS, Calcium montmorillonite; UPSN, calcium montmorillonite Uniform particle size Novasil; FB_1_, Fumonisin B_1_; UFB_1_, Urinary FB_1;_AFB_1_, Aflatoxin B_1_; AFM_1_, Aflatoxin M_1_; Bw, Body weight*.

Because natural clay mineral adsorbents are considered GRAS by the US FDA (2015), they could be applied effectively and economically in the food and feed industries and several clay minerals have been proven to be acceptable for commercial uses [US FDA, GRAS substances evaluated by the Select Committee on GRAS substances (SCOGS); He and Zhou, 2010]. However, application of clay minerals often requires high levels to be included into animal feed; interaction of natural clays with food- and gut-based nutrients remains unclear; and the possibility of accumulation of dioxin (a toxic trace component in montmorillonite) in animals remains a concern.

### Microbial transformation of the fumonisins

Development of control methods to detoxify the fumonisins through transformation should be directed toward deamination of the free amino group at C-2 and hydrolysis of the ester bonds at C-14 and C-15 (Gelderblom et al., 1993). Microorganisms capable of transforming FB_1_ to less toxic end products include *Exophiala spinifera* ATCC 74269, *Rhinocladiella atrovirens* ATCC 74270, Bacterium ATCC 55552, and *Sphingopyxis macrogoltabida* MTA144 (Duvick et al., 1998a,b; Blackwell et al., 1999; Heinl et al., 2010). Transformation of FB_1_ by the black-yeast *E. spinifera* was mainly achieved through decarboxylation by inducible extracellular esterase enzymes and amino oxidases converting hydrolysed fumonisin (HFB_1_) to unknown end products. Degradation by Bacterium ATCC 55552 and *S. macrogoltabida* MTA144 is achieved through de-esterification by carboxylesterases and subsequent deamination of HFB_1_ by aminotransferases, with the formation of 2-keto HFB_1_ (Heinl et al., 2010; Hartinger et al., 2011). The microbial gene sequences coding for these enzymes were determined by employing degenerate polymerase chain reaction (PCR) primers, inverse PCR and gene walking techniques. Carboxylesterase (FumD) and aminotransferase enzymes (FumI) of *S. macrogoltabida* MTA144 and Bacterium ATCC 55552 were expressed in *Pichia pastoris* and *E. coli*, respectively, by employing episomal pET-3a vectors. Production of the recombinant enzymes were induced in liquid cultures by isopropyl-beta-D-thiogalactopyranoside, where after degradation of FB_1_ and HFB_1_ was demonstrated with the recombinant culture supernatant as well as with purified enzyme preparations. HFB_1_ prepared through enzymatic transformation by FumD carboxylesterases exhibited considerable less toxicity than FB_1_ when evaluated in a pig intestine model as indicated by the modified sphinganine/sphingosine ratios in the liver and plasma, modified intestinal immune response, and absence of hepatotoxicity and impaired intestinal morphology (Oswald et al., 2012). Although, certain of these technologies are considered safe for humans, animals and the environment by the European Food Safety Authority (EFSA), applications of microbial enzymes are presently mainly directed toward the animal feed industry (Duvick et al., 1998b, 2003; Moll et al., 2011). Recombinant enzymes are mass produced in a bioreactor and are applied during storage and food-processing to incorporate into animal feed and act in the intestinal tract of animals, or for treatment of grains in the form of a wash, additive or spray. Other post-harvest methods involving microbial transformation include the engineering of ruminal organisms and supplementation to feed in the form of a probiotic inoculant.

### Commercialization of biological methods of control

The lack of effective and environmentally safe chemical control methods against fungal growth and mycotoxin production in food crops has led to investigations into biologically safe alternatives to prevent these contaminants from entering the food chain (Beekrum et al., 2003). Biological pesticides and methods involving natural resources such as plants, microorganisms, genetic factors thereof, and clay minerals are popular alternatives being evaluated for control of mycotoxigenic fungi in grains (Alabouvette et al., 2009). *Fusarium* growth and fumonisin production pre-harvest and post-harvest are effectively reduced by several natural and biological methods involving plant material, microorganisms and minerals, as evident by the extensive research done on this subject in recent years.

Several commercial products for biological control of *Fusarium* diseases and the fumonsins have been developed for application alone, in combination or as part of an integrated control strategy. Products containing biocontrol microorganisms are mainly aimed at application as seed and soil treatments as outlined by Fravel et al. (1998) and Kahn (2013):

- “Fusaclean” and “Biofox C” (non-pathogenic *F. oxysporum* for control of *F. oxysporum* and *F. verticillioides* in a variety of vegetables).
- “Epic” and “Kodiak” (*B. subtilis* for control of *Fusarium* in cotton and legumes).
- “Intercept” (*Pseudomonas cepacia* for control of *Fusarium* in maize, vegetables and cotton).
- “Mycostop” (*Streptomyces griseoviridis* for control of *Fusarium* in ornamental and vegetables crops).
- T-22G and T-22HB (*Trichoderma harziatum* for control of *Fusarium* in grains, soya, cotton and vegetables).
- “Biofungus” (*Trichoderma* spp. for control of *Fusarium* in citrus and pome fruit).
- “Blue circle” (*Burkholderia cepacia*) for control of *Fusarium* in vegetables).
- “Deny” (*B. cepacia* for control of *Fusarium* in a variety of grain crops).
- “Cedomon” and “Cerall” (*Pseudomonas chlororaphis* for control of *Fusarium* in wheat, rye and triticale).
- Commercial GRAS products developed from clay minerals include Novasil ® and Nevalite® (calcium montmorillonite) (Robinson et al., 2012).
- Fumzyme® (Biomin, Austria) was developed from the carboxylesterase enzyme of *S. macrogoltabida* (Heinl et al., 2010).

Although, there is an increased interest in biological control methods, much effort is put into details of natural compounds capable of controlling fungal growth and mycotoxins *in vitro*. However, the growing knowledge base on this subject should be further developed for application *in planta* and in the field pre-harvest, post-harvest, and during storage and food-processing. In order to develop the available information into appropriate methods for application *in planta* and in the field, there are many economic and technological hurdles to overcome. The effectiveness of antioxidants, essential oils, phenolic compounds and combinations for example, has been demonstrated at laboratory scale, and bioactivity in the vapor phase makes it promising as fumigant for protection of grains on the field immediately after harvest or during storage (Chulze, 2010). However, evaluation studies in grains are limited due to cost implications and the inhibitory effect in maize generally achieved with higher concentrations than in synthetic media, because of possible matrix interference and reduced bioavailability relating to distribution on kernel surfaces and penetration into the pericarp (Torres et al., 2003; Samapundo et al., 2007). In certain cases, high concentrations of phenolic compounds could also affect the sensory quality of the maize. Certain antioxidants such as BHA and PP, clay minerals, and plant extracts are considered GRAS, making it very promising for biocontrol purposes. Mixtures of antioxidants or combinations with other food preservatives (i.e., benzoic and sorbic acids) could further enhance the antifungal efficacy (Reynoso et al., 2002).

Even though biologically based treatments most likely will have a reduced effect than chemical methods on the desired nutritional value, quality, safety, or sensory attributes of foods and feed and impact on the environment, compliance to food safety assessment guidelines, such as those prescribed by the European Network on Safety Assessment of Genetically Modified Food Crops (ENTRANSFOOD) and the FAO/WHO, have to be met (He and Zhou, 2010). Assessments could include compositional analyses of key components of treated food including nutrients, micronutrients, and predictable secondary metabolites; assessment of possible toxicity, allergens; potential environmental impact; long-term nutritional impact; influence of food/feed processing; potential dietary intake and change in dietary pattern. While there are several opportunities for further exploring and developing biological control methods for *Fusarium* growth and fumonisins, each method has its own challenges. However, an integrated approach, involving good agricultural management practices, HACCP models and storage management, together with appropriately selected biologically based microbial treatments, mild chemical and physical treatments could reduce *Fusarium* diseases and fumonisins effectively pre- and post-harvest (da Cruz Cabral et al., 2013).

### Practical and culturally acceptable methods for mycotoxin reduction—approaches in sub-Saharan countries

Methods for prevention of chronic exposure to the fumonisins, particularly in low socio-economic rural subsistence farming communities, remain critically important. In developed countries high standards of the major food suppliers and retailers are upheld and the regulatory controls deter the importation and marketing of seriously contaminated products. In developing countries only a limited number of countries have legislative maximum levels for fumonisins, and implementation thereof is often poor. In rural subsistence farming communities, legislation is not applicable and with continued pressure on food security, an increased **mycotoxin exposure** on a daily basis is the norm. In addition, due to the stringent mycotoxin standards in developed countries, the best-quality food products are normally exported resulting in highly contaminated foods being utilized domestically which increases the risk of mycotoxin exposure and the associated adverse health effects (Pitt et al., 2012). High risk population groups include rural communities and/or subsistence farmers heavily reliant on maize as their staple diet. Although, commercial maize is contaminated with lower levels, daily exposure could be a risk factor for disease development in impoverished communities.

In developing countries, where resources are limited and sophisticated technologies are lacking, the importance of cost-effective and simple intervention methods, predominantly at population level, has been emphasized. In this regard, culturally acceptable simple, practical and biologically based methods of reduction are relevant, as a last line of defense in rural subsistence farming communities exposed to high levels of the fumonisins in their staple diet. Effective reduction has been demonstrated with hand sorting, flotation, washing, dehulling of maize kernels and combinations thereof *in vitro* and in field studies (Table 5). Dehulling and shelling of maize are common practices in West-Africa (Fandohan et al., 2006), with the removal of the pericarp an effective way to reduce mycotoxin contamination (Sydenham et al., 1994; Bullerman and Bianchini, 2007; Burger et al., 2013). The effectiveness of hand-sorting of maize by removing visibly infected and damaged kernels, resulting in a significant reduction of fumonisins has been demonstrated in several African countries, including Benin (Fandohan et al., 2005), Nigeria (Afolabi et al., 2006), Tanzania (Kimanya et al., 2008), South Africa (Van der Westhuizen et al., 2010), and Malawi (Matumba et al., 2015). In South Africa a simple, practical and culturally acceptable hand-sorting and washing intervention method was developed and implemented for reduction of fumonisin exposure in a subsistence maize-farming community (Van der Westhuizen et al., 2010, 2011b). The efficacy of the maize kernel wash method could possibly be further enhanced by incorporating clay minerals or fumonisin detoxifying enzymes. Advantages of interventions involving practical methods usually take the form of improved health outcomes rather than market outcomes (Wu and Khlangwiset, 2010a,b). Public health interventions should be culturally acceptable; be implemented through educational campaigns; and must have financial and infrastructural support to be feasible in remote rural areas where they are most needed. Sustainability of these reduction strategies is, however, dependent on the available maize supply (food security), as well as the socio-economic status and education of a community.

**Table 5:** **Practical and culturally acceptable methods of mycotoxin reduction for rural subsistence farming communities exposed to high levels of fumonisins in their staple diet (***in vitro***, field- and intervention studies)**.

**Method of mycotoxin reduction**	**Test system with details of experimental model**	**Reduction criteria**	**Application**	**Reference(s)**
Hand-sorting of maize	Occurrence of *Fusarium* spp. and mycotoxins in Nepalese maize and wheat and the effect of traditional processing methods on mycotoxin levels. Field study: Study area: Kathmandu, Nepal; Maize samples: purchased at a market in Kathmandu; the samples contained large amounts of visibly diseased kernels; Hand-sorting: participants: four trained plant pathologists, three untrained urban women, five women from smallholder farms in the Lamjung district of Nepal; removal of visibly diseased kernels; maximizing the recovery of the starting sample; Fumonisin and DON levels: immunoassay or HPLC	Field study: Hand-sorting: all participants were able to produce a product with acceptable fumonisin and DON levels; large differences between participants with regards to maximizing the recovery of the starting sample: plant pathologists and two rural women (86% recovery), three untrained urban women and three rural women (49% recovery); Fumonisin and DON levels: maize samples prior to hand-sorting: >1000 ng toxin/g maize; maize samples after hand-sorting: < 1000 ng toxin/g maize	Hand-sorting is economically viable for populations with limited food resources; most of the starting material should be recovered in the cleaned product; educational campaigns to raise awareness among Nepalese consumers on the occurrence of mycotoxins in maize and the efficacy of hand-sorting methods	Desjardins et al., 2000
Hand-sorting, winnowing, washing, crushing, and dehulling of maize	Fate of aflatoxins and fumonisins during the processing of maize into food products in Benin. *In vitro:* Impact of sorting, winnowing, washing, and crushing of maize on fumonisin levels in maize intended for the preparation of traditional maize-based food: Sorting: removal of visibly moldy, insect damaged and broken kernels; Winnowing (complementary to sorting): removal of impurities from sorted maize by collecting maize in a metallic tray, throwing contents into the air and allowing impurities and broken kernels to be blown away; Maize washing (complementary to sorting and winnowing): maize to water ratio 1:2 (w/v); hand rubbing of kernels (15 min); removal of floating grains and impurities; Crushing and dehulling (complementary to sorting, winnowing and washing) (removal of pericarp and embryo): crushing with plate disc mill; sieving to obtain separately grits, hulls and fine fractions; hand washing of grits (10-15 min); soaking in water (2 h) [grits to water ratio 1:3 (w/v)]; Total fumonisin levels in fractions: ELIZA (VICAM)	*In vitro:* Sorting and winnowing: 68.75% reduction in total fumonisin content of maize; total fumonisin levels were high in the moldy and damaged kernels; Maize washing (complementary to sorting and winnowing): additional 15.34% reduction in total fumonisin content of maize; total fumonisin levels were high in the upper floating grain fractions; significant amount of fumonisins detected in washing water; Crushing and dehulling (complementary to sorting, winnowing and washing): significant (*P* < 0.05) reduction of total fumonisin levels; no fumonsins detected in washed grits	Reduction of fumonisins in maize intended for traditional food preparation in rural subsistence farming households: systematic cleaning of maize, involving sorting and washing, performed prior to preparation of maize-based food	Fandohan et al., 2005
Mechanical shelling and dehulling of maize	Impact of mechanical shelling and dehulling on *Fusarium* infection and fumonisin contamination in maize. *In vitro:* Impact of shelling methods on *Fusarium* and fumonisin contamination: shelling by hand, handle-operated sheller, two commercial motorized shellers; Impact of dehulling on fumonsin contamination: dehulling with attrition disk mill, two commercial motorized dehullers; Determination of moisture content, percentage of damage cause by the method, mean *Fusarium* population (cfu.g^−1^) and total fumonisin levels: ELIZA (VICAM)	*In vitro*: Impact of shelling methods on *Fusarium* and fumonsin contamination: all mechanical shelling methods caused damage to maize kernels; *Fusarium* colony count highest (*P* < 0.05) in maize shelled with mechanical sheller; *Fusarium* colony count positively and significantly correlated with percentage of kernel damage (*r* = 0.6; *P* < 0.01); total fumonisin levels the highest (*P* < 0.01) in maize shelled with mechanical shellers; fumonisin levels positively and significantly correlated with both the percentage of damage caused by shelling method (*r* = 0.6; *P* < 0.01) and the *Fusarium* colony count (*r* = 0.7; *P* < 0.01); Impact of dehulling on fumonsin contamination: the total fumonisin levels in maize was significantly reduced (57–65% reduction; *P* < 0.01) by all the dehulling methods tested	Promotion of dehulling for reduction of mycotoxins in maize; introduction of dehulling methods in African countries where it is still uncommon; selection of appropriate shelling methods to limit kernel damage and reduce mycotoxin contamination	Fandohan et al., 2006
Hand-sorting of maize	Effect of sorting on incidence and occurrence of fumonisins and *Fusarium verticillioides* on maize from Nigeria. Analyses of field samples: Collection of maize samples in the Kaduna state of Nigeria; hand-sorted “good” and “poor” quality maize were collected from farmers' stores; Incidence of *F. verticillioides* in maize: mycological analyses: isolation, identification and quantification of *Fusarium* spp.; maize kernels plated out on semi-selective *Fusarium* medium peptone-pentachloronitrobenzene agar; single-spores transferred to carnation leaf agar for identification; identification with standard morphological criteria; Confirmation of the identity of selected *F. verticillioides* strains: amplified fragment length polymorphisms; Fumonisin levels in maize: ELIZA	Analyses of field samples: “Good” quality maize contained < 7% visibly diseased kernels; “Poor” quality maize contained mostly >30% visibly diseased kernels; Fumonisin levels in maize: “good” quality maize contained low fumonisin levels (0 - 0.2–3.7 μg/g maize); “poor” quality maize contained fumonisin levels 1.4–110 μg/g maize; fumonisin levels significantly (*P* < 0.0001; *r* = 0.697) correlated with the percentage of visibly diseased kernels; *F. verticillioides* recovered from every sample that was positive for fumonisins	An appropriate method for reducing fumonisin exposure in rural subsistence farming communities of West Africa; only effective if “good” quality maize is consumed alone and “poor” maize discarded; educational and awareness campaigns should be performed in rural Africa: information on hand-sorting as reduction method and the health risks of using sorted moldy maize as animal feed	Afolabi et al., 2006
Hand-sorting of maize	Co-occurrence of fumonisins with aflatoxins in home-stored maize for human consumption in rural villages of Tanzania. Analysis of field samples: Study area: rural subsistence farming communities in high maize production regions of Tanzania; Sampling of maize: shelled and unshelled maize for human consumption from households and stores; 5–6 months after harvest; FB_1_ and FB_2_ levels in maize samples: HPLC; Determination of the percentage of defective kernels; Collection of information from the community: questionnaires on practices with regards to the type of staple food and the handling, storage, sorting and discarding of maize; involving heads of households	Analysis of field samples: Eighty-eight percent of maize samples contained defective kernels at levels above 7% (maximum limit recommended by the Codex Alimentarius Commission for maize or Corn); FB_1_ and FB_2_ levels in maize samples: positive correlation between fumonisin levels and the extent of defective kernels (*r* = 0.39); Maize containing less than 7% defective kernels contained relatively low contamination of fumonisins, suggesting that sorting of maize before consumption is an important measure for reduction	Reduction of fumonisin exposure in rural subsistence maize farming communities at risk: sorting of maize prior to storage; implementation of sorting methods by farmers and households in affected rural areas; educational and awareness campaigns on the health risks of using sorted moldy maize as animal feed or as raw material for beer making	Kimanya et al., 2008
Hand-sorting and washing of maize	Simple intervention method to reduce fumonisin exposure in a subsistence maize-farming community in South Africa. Field study: Study area: rural subsistence farming communities in the Centane magisterial district of the Eastern Cape Province of South Africa; Participants: females who prepare traditional maize-based food from home-grown maize; Baseline phase of study: Preparation of maize-based stiff porridge by participants according to their customary practices; consumption of porridge (2x 0.5 kg portions for two consecutive days); Assessment of porridge intake: 24 h dietary recall questionnaire by utilizing full-scale photographs of portions; Intervention phase of study: Hand-sorting: training of participants by field workers demonstrating the removal of infected and damaged kernels with the aid of photographs; sorting of a 4 kg maize kernel batch by participants under the supervision of the field workers; Washing of maize: demonstration of a 10 min maize water washing procedure: 5 min hand agitation and 1-min agitation prior to the 10 min end point; washing of good sorted kernels by participants under supervision of the field workers; Drying of subsamples of sorted and washed kernels; Preparation of traditional stiff porridge by field workers; consumption of a weighed portion (0.5 kg) by each participant; extra portion of stiff porridge supplied to participants; Assessment of porridge intake: 24 h dietary recall questionnaire; Determination of total fumonisin levels in sorted and washed maize, and in maize porridge from the baseline and intervention phases: HPLC; Determination of fumonisin exposure: total fumonisin levels in the stiff porridge consumed by each participant during the baseline and intervention phases of the study	Field study: Two-step intervention procedure (hand-sorting and washing of maize): 84% reduction of total fumonisin levels in maize batches; 65% reduction of total fumonisin levels in maize porridge; 62% reduction in fumonisin exposure	An effectively implemented simple, practical and culturally acceptable intervention method for reduction of fumonisin exposure in rural subsistence maize farming communities exposed to high levels of fumonisins in their staple diet	Van der Westhuizen et al., 2010
Hand-sorting and washing of maize	FB_1_ as a urinary biomarker of exposure in a maize intervention study among South African subsistence farmers. Field study: Study area: rural subsistence farming communities in the Centane magisterial district of the Eastern Cape Province of South Africa; Baseline and Intervention phases of the study: Performed similar to the study described above Van der Westhuizen et al., 2010; Urine collection: morning first-void urine collections approximately 12 h after the participants consumed the last meal; PDI assessment: assessment of porridge intake: 24 h dietary recall questionnaire; individual fumonisin PDI assessed as FB_1_ level in porridge (dry weight) consumed by each participant during the baseline and intervention phases of the study; FB_1_ levels in maize and porridge: HPLC analyses; Determination of UFB_1_ biomarker in urine: LC-MS analyses; determination of the percentage UFB_1_ excretion	Intervention study: Hand-sorting and washing of maize: significant (*P* < 0.05) reduction in FB_1_ levels (84% reduction); PDI assessment: Mean PDI of FB_1_ at baseline significantly (*P* < 0.05) reduced with 62% following the intervention: before the intervention PDI levels of 71% participants exceeded JECFA recommended PMTDI level for FB_1_; following the intervention only 53% of participants exceeded the recommended PMTDI level; Urine: UFB_1_ in urine was reduced with 52% (*P* = 0.02) following the intervention; normalization with UFB_1_C indicated a 41% reduction (*P* = 0.06);	Simple, practical and culturally acceptable intervention for reduction of fumonisin exposure in rural subsistence farming communities exposed to high levels of fumonisins in their staple diet; utilization of this biomarker will improve assessment of fumonisin exposure, contribute to assessment of possible health impacts of fumonisin exposure and permit evaluation of intervention strategies to reduce fumonisin exposure; future interventions could be expanded to larger number of both male and female participants including children	Van der Westhuizen et al., 2011a
Laboratory-optimized hand-sorting and washing of maize	Optimising sorting and washing of home-grown maize to reduce fumonisin contamination under laboratory-controlled conditions. *In vitro*: Study area: rural subsistence farming communities in the Centane magisterial district of the Eastern Cape Province of South Africa; Questionnaires on customary sorting and washing of maize: focus groups; females who traditionally prepare maize meals; interviews with field workers; Maize: obtained from rural subsistence farming households; Hand-sorting and washing procedures: as described above Van der Westhuizen et al., 2010; Effect of water temperature (5 min wash): 25 and 40°C; Effect of wash duration (25°C): 5, 10, 30 and 60 min; Mycological analyses: determination of percentage kernels infected; determination of the frequencies of *Fusarium* and *Stenocarpella* species Total fumonisin levels in maize samples: HPLC	*In vitro:* Questionnaires on customary sorting and washing of maize: hand-sorting directly after harvest; moldy cobs are discarded, but in certain cases used for food preparation; winnowing and removal of plant debris; washing of good kernels prior to cooking; 30% of focus groups use quick ambient temperature water rinse; 35% do a 3–8 min water wash; 10% do a 3–5 h wash; 25% used warm water; 70% discard wash water in the field; 30% give wash water to farm animals; Mycological analyses: kernels mostly infected by *Fusarium verticillioides* (17%), *Fusarium graminearum* (9%), *Fusarium subglutinans* (5%) and *Fusarium anthophilum* (1%); Hand-sorting: 71±18% reduction in total fumonisin levels; Effect of temperature (5 min wash): additional 4±6% reduction in total fumonisin levels at 40°C; Effect of wash duration (25°C): additional 13±12% reduction in total fumonisin levels after 10 min wash	Method recommended for reduction of fumonisin exposure in rural subsistence maize farming communities: removal of infected/damaged kernels from maize followed by a 10 min ambient temperature water wash, with sufficient water to cover maize; maize wash water needs to be discarded	Van der Westhuizen et al., 2011b
Hand-sorting, flotation/washing, dehulling of maize and combinations thereof	Effectiveness of hand-sorting, flotation/washing, dehulling and combinations thereof on the decontamination of mycotoxin-contaminated white maize. Analysis of field samples: Maize: Visually moldy white maize kernels purchased from a local market in the Chikwawa district of Malawi; winnowing and mixing; Three factorial design experiment with variables sorting, flotation/washing and dehulling in 8 independent experiments (including the control): Hand-sorting: removal of visibly moldy kernels; Removal of moldy kernels by flotation and washing of non-floating kernels: maize to water ration 1:2 (w/v); stirred by hand and allowed to stand for 5-10 s; removal of top floating fraction; repetition of procedure until all floating kernels and particles were removed; Washing of non-floating kernels: maize to water ratio 1:2 (w/v); 2x 1 min wash; Dehulling: untreated maize and maize without the fractions removed through hand-sorting and flotation (4.5 kg); addition of water (200 ml); dehulling with a mortar and pestle; manual winnowing; FB_1_, FB_2_ and FB_3_ levels in maize samples: LC-MS/MS	Analysis of field samples: Fumonisins are concentrated in moldy, broken and discolored maize kernels; Hand-sorting had the largest effect among the single methods, followed by dehulling and flotation (in this order); Percentage reduction of fumonisin levels in maize: Hand-sorting: 91.6–95.7% Dehulling: 85.2–90.3% Flotation: 67–77.8% Percentage reduction of fumonisin levels in maize after combined treatments: Flotation^*^Hand-sorting: 63.8–76.5% Flotation^*^Dehulling: 60.7–70.4% Hand-sorting^*^Dehulling: 79.2–87.3% Flotation^*^Hand-sorting^*^Dehulling: 58.6–61.9%; Hand-sorting of maize resulted in much lower mass loss than dehulling	Reduction of fumonisin exposure in rural subsistence farming communities at risk: hand-sorting of maize kernels proofed very effective and is recommended as last line of defense; dehulling might not be necessary if hand-sorting is thoroughly applied; integration of hand-sorting into the maize production and utilization chain; campaigns by governments and relevant developing partners to raise public awareness and promote the hand-sorting method	Matumba et al., 2015

FB_1_, Fumonisin B_1_; FB_2_, Fumonisin B_2_; FB_3_, Fumonisin B_3_; UFB_1_, Urinary FB_1_; UFB_1_C, urinary FB_1_creatinine; DON, deoxynivalenol; PDI, Probable daily intake; PMTDI, Provisional maximum tolerable daily intake; JECFA, The Joint FAO/WHO Expert Committee on Food Additives. ^*^, Indicates combined treatments.

## Author contributions

Dr. JA, Wrote article; Prof. WG, Coordinated and assisted in writing article; Prof. WV, Assisted in writing article.

### Conflict of interest statement

The authors declare that the research was conducted in the absence of any commercial or financial relationships that could be construed as a potential conflict of interest.
